# Co-Aggregation and Parallel Aggregation of Specific Proteins in Major Mental Illness

**DOI:** 10.3390/cells12141848

**Published:** 2023-07-13

**Authors:** Bobana Samardžija, Maja Juković, Beti Zaharija, Éva Renner, Miklós Palkovits, Nicholas J. Bradshaw

**Affiliations:** 1 Department of Biotechnology, University of Rijeka, 51000 Rijeka, Croatia; bobana.samardzija@biotech.uniri.hr (B.S.); maja.jukovic@biotech.uniri.hr (M.J.); beti.zaharija@biotech.uniri.hr (B.Z.); 2 Human Brain Tissue Bank & Laboratory, Semmelweis University, 1094 Budapest, Hungary; renner.eva@med.semmelweis-univ.hu (É.R.); palkovits.miklos@med.semmelweis-univ.hu (M.P.)

**Keywords:** insular cortex, mental illness, post mortem brain tissue, protein aggregation, proteinopathy, suicide

## Abstract

Background: Disrupted proteostasis is an emerging area of research into major depressive disorder. Several proteins have been implicated as forming aggregates specifically in the brains of subsets of patients with psychiatric illnesses. These proteins include CRMP1, DISC1, NPAS3 and TRIOBP-1. It is unclear, however, whether these proteins normally aggregate together in the same individuals and, if so, whether each protein aggregates independently of each other (“parallel aggregation”) or if the proteins physically interact and aggregate together (“co-aggregation”). Materials and methods: Post mortem insular cortex samples from major depressive disorder and Alzheimer’s disease patients, suicide victims and control individuals had their insoluble fractions isolated and tested by Western blotting to determine which of these proteins are insoluble and, therefore, likely to be aggregating. The ability of the proteins to co-aggregate (directly interact and form common aggregate structures) was tested by systematic pairwise expression of the proteins in SH-SY5Y neuroblastoma cells, which were then examined by immunofluorescent microscopy. Results: Many individuals displayed multiple insoluble proteins in the brain, although not enough to imply interaction between the proteins. Cell culture analysis revealed that only a few of the proteins analyzed can consistently co-aggregate with each other: DISC1 with each of CRMP1 and TRIOBP-1. DISC1 was able to induce aggregation of full length TRIOBP-1, but not individual domains of TRIOBP-1 when they were expressed individually. Conclusions: While specific proteins are capable of co-aggregating, and appear to do so in the brains of individuals with mental illness and potentially also with suicidal tendency, it is more common for such proteins to aggregate in a parallel manner, through independent mechanisms. This information aids in understanding the distribution of protein aggregates among mental illness patients and is therefore important for any future diagnostic or therapeutic approaches based on this aspect of mental illness pathology.

## 1. Introduction

Major depressive disorder (MDD), bipolar disorder and schizophrenia are all severe and often chronic mental illnesses, which have profound influences on patients, their families and society in general. The underlying pathophysiology of these conditions is partially understood, in large part due to advances in uncovering genetic risk factors. However, these studies present a highly heterogenous picture, with many risk factors of small effect accounting for a proportion of heritability and the remaining risk assumed to come from rarer mutations [1,2,3]. As a supplement to this approach, we and others have proposed instead to consider the role of proteinopathy in chronic mental illnesses [4]. Specifically, in partial analogy to how specific proteins form misfolded or unfolded aggregates in the brains of patients with neurodegenerative disease, similar aggregates, of differing proteins, may also exist in the brains of at least some patients with chronic mental illness. Unlike in neurodegenerative diseases, we do not expect these aggregates to be neurotoxic, given that neuronal loss (on the scale seen in neurodegenerative disease) is not a characteristic of major mental illnesses.

Studies investigating aggregation of these proteins in mental illness generally use insolubility as an indicator of aggregation [4]. Protein aggregates are larger agglomerates with either an incorrect or random structure, and as a result are normally insoluble in cells and common experimental systems. By taking homogenized brain samples, purifying only the more insoluble protein fraction and testing by Western blotting, it can be determined whether a specific protein is insoluble in the original brain sample [5,6]. Should a normally soluble protein be found in the insoluble fractions of patient brain samples specifically, this then provides a strong argument for this protein aggregating in the associated illness. Such approaches have detected Disrupted in Schizophrenia 1 (DISC1), dysbindin 1, Collapsin Response Mediator Protein 1 (CRMP1) and TRIO Binding Protein isoform 1 (TRIOBP-1) as insoluble in subsets of patients with schizophrenia, bipolar disorder and/or MDD [5,6,7,8,9,10], while Neuronal PAS protein 3 (NPAS3) is implicated through an aggregation-inducing mutation [11,12]. In all cases, insoluble protein has also been detected in mammalian cell culture models and found to be equivalent to visible protein aggregates in the cell body [6,7,9,10,12,13]. The fact that these events are not diagnosis specific, however, raises the interesting possibility that protein aggregation, both generally or of these specific proteins, may be a common feature in mental illness. To date, however, sufficient samples sizes have not been available to determine whether these aggregation events correspond to specific subtypes or symptoms of these illnesses.

**Figure 1:**
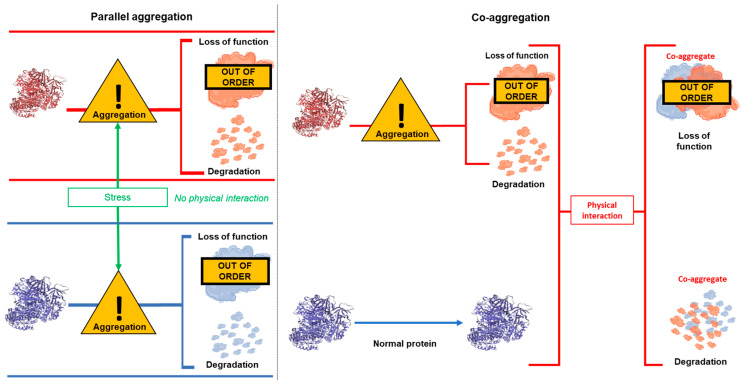
Illustration of the concepts of parallel aggregation and co-aggregation. Parallel aggregation occurs when some form of stress (for example oxidative stress or genetic mutation) causes two proteins to aggregate. The stress may be common to both proteins or separate, but regardless leads to both proteins co-existing as physically distinct aggregates. Co-aggregation occurs when stress causes one protein to aggregate. This protein then physically interacts with another, previously non-aggregating, protein, causing it also to misfold and aggregate. In all instances, the cell would be expected to attempt to clear aggregating proteins, for example through the proteasome, but visible aggregates still form when these systems are not able to deal with the level of misfolded/aggregating protein present in the cell.

Another interesting finding from previous work is that in some, but not all, cases, insoluble DISC1 co-exists in the same brain samples as insoluble dysbindin-1 and CRMP1 [7,9]. Subsequent analysis then showed that DISC1 could directly bind to and induce aggregation of both of these proteins, in a process of “co-aggregation”. Other combinations of proteins implicated in major mental illness have not yet been tested in the same way. It is therefore unclear whether, in general, these proteins each aggregate alone, possibly in distinct patient populations, or co-aggregate together as part of a more general proteinopathy. This latter idea is supported by observations that the ubiquitination and proteasome system of the cell has been shown to be generally less functional in schizophrenia patients, while total insoluble protein is higher [14,15,16]. It is therefore also possible that common underlying causes or stresses could lead to multiple proteins aggregating in the same brain, or even the same neuron, without the proteins physically interacting with each other, in a process of “parallel aggregation”. The concepts of parallel and co-aggregation are also illustrated in Figure 1.

Therefore, here we investigate the potential for co-aggregation and parallel aggregation in major mental illness, using two distinct approaches: investigation of protein insolubility in a set of human brain samples and systematic investigation of co-aggregate forming potential in human neuroblastoma cells.

## 2. Materials and Methods

### 2.1. Human Brain Tissue

Collection, storage and distribution of human brain tissue were approved by the Committee of Science and Research Ethics of the Ministry of Health of Hungary (No. 6008/8/2002/ETT) and the Semmelweis University Regional Committee of Science and Research Ethics (No. 32/1992/TUKEB). Use of brain tissue for this project was approved by the Ethical Committee of the University of Rijeka, Department of Biotechnology (23 October 2018). All work was performed in accordance with the Declaration of Helsinki and all national and European laws. Informed familial consent or legal permission was acquired before collecting each sample. As part of the Hungarian Lenhossék program, brains were collected with short post mortem delays (2–10 h), and samples of various brain regions were isolated using the “micro-punch” technique [17,18]. Brain samples were then frozen and stored at −80 °C. For this work, such samples were used from the insular cortices of victims of suicide (*n* = 15) and control individuals (*n* = 11), as well as patients with MDD (*n* = 4) and Alzheimer’s disease (*n* = 3). None of the control individuals had a history of mental illness. Clinical data are only available for some of the suicide victims, and the sample can be assumed to contain both individuals with and without depression (diagnosed or undiagnosed). Levels of NPAS3 in this cohort have been investigated previously, and demographic information can be found in that publication [13]. All tissue donors were Hungarian, with 44% being female and 56% male.

### 2.2. Insolubility Assay

Samples of brain homogenate had their insoluble protein fraction purified as described previously [6]. Briefly, samples of 10% brain homogenate are solubilized, treated with DNaseI and then subjected to a number of ultracentrifugation steps, after which the soluble (liquid) protein fraction is discarded. The insoluble pellet is then resuspended and centrifuged again. Buffers variously include high salt, high sucrose and detergents to isolate only the most insoluble protein in the sample, which is predicted to contain protein aggregates. Cell lysates had their insoluble proteins isolated using a similar technique, also described previously [13].

### 2.3. Antibodies

Primary antibodies were purchased against β-actin (Origene, Rockville, MD, USA, OG-TA811000), CRMP1 (ProSci, Poway, CA, USA, 3625), DISC1 (Thermo Fisher Scientific, Weltham, MA, USA, 40-6800), Flag (Merck, Darmstadt, Germany, F1804), GFP (Merck, G6795) and TRIOBP-1 (Atlas Antibodies, Stockholm, Sweden, HPA019769). Secondary antibodies were from Thermo Fisher Scientific (31430, 65-6120 and A11037).

### 2.4. Plasmids

Vectors encoding human CRMP1 [9] and TRIOBP-1, both full length and fragments [19], were gifts from Prof. Dr. Carsten Korth (Heinrich Heine University, Düsseldorf, Germany), with CRMP1 then being subcloned into pENTR1A no ccDB [20] (Dr. Eric Campeau, AddGene clone 17398, Watertown, MA, USA). Vectors encoding NPAS3 [13] and a TRIOBP-1 aggregation-resistant mutant [6] were described previously. Gateway entry vectors encoding full length human DISC1 and NPAS3 came from the ORFeome Collaboration [21,22] (DNASU Plasmid Repository, clones HsCD00516321 & HsCD00080332, Tempe, AZ, USA). Entry vectors were transferred into pDEST-CMV-N-EGFP [23] (Prof. Robin Ketteler, AddGene clone 122842) and/or pdcDNA-FlagMyc (B. Janssens, BCCM/LMBP Plasmid Collection, clone LMBP 4705, Zwijnaarde, Belgium) using LR Clonase II (Thermo Fisher Scientific). More details of the plasmids and primers used are in Supplementary Tables S1 and S2. All plasmids were confirmed by sequencing, and amino acid sequences of proteins expressed can be found in Appendix S1 of the Supplementary Material.

### 2.5. Cell Culture

HEK293 human kidney cells (American Type Culture Collection, Manassas, VA, USA, CRL-1573) were cultured in D-MEM (Thermo Fisher Scientific), supplemented with 5% HyClone Cosmic Calf serum (Cytiva, Marlborough, MA, USA), penicillin and streptomycin (Pan-Biotech, Aidenbach, Germany). Plasmids were transfected into cultured cells using Metafectene (Biontex, Munich, Germany) according to manufacturer’s protocols. SH-SY5Y human neuroblastoma cells (Deutsche Sammlung von Mikroorganismen und Zellkulturen, Braunschweig, Germany, ACC 209) were cultured in D-MEM/F-12, supplemented with 5% fetal calf serum (both Thermo Fisher Scientific), non-essential amino acids, penicillin and streptomycin (Pan-Biotech). Plasmids were transfected into cultured cells using Metafectene Pro (Biontex) according to manufacturer’s protocols.

### 2.6. Western Blotting

Samples were denatured in 156 mM Tris pH 6.8/5% SDS/20 mM DTT/25% glycerol with bromophenol blue for 5 min at 95 °C and then separated on bis-acrylamide gels. Gels were transferred to PVDF membranes (Macherey-Nagel, Düren, Germany) using a Transblot Turbo system (Bio-Rad, Hercules, CA, USA) and transfer was confirmed by staining with 0.5% Ponceau S/2% acetic acid. Membranes were blocked for 1 h at room temperature in PBS/0.05% Tween-20/5% milk powder, and then stained overnight at 4 °C using primary antibodies diluted in the same buffer. Membranes were washed 4 times over 30 min with PBS/0.05% Tween-20 and then stained with secondary antibodies (1 h, room temperature, 10,000-fold dilution), in the same buffer. Membranes were then washed 4 times over 30 min with PBS/0.05% Tween-20, and the signal revealed using ECL (Thermo Fisher Scientific). The signal was detected and quantified using a ChemiDoc MP Imaging System and ImageLab 5.2 software (Bio-Rad).

### 2.7. Immunocytochemistry and Microscopy

Cells on glass coverslips were fixed with PBS/4% paraformaldehyde for 15 min and then permeabilized with PBS/0.5% Triton X-100 for 10 min. Cells were blocked with PBS/10% goat serum (Merck) for 30 min and then stained with primary antibody in the same media for 2–4 h. Cells were then washed 3 times (5 min each) with PBS and stained with secondary antibodies, at 500-fold dilution, and DAPI (Merck) in PBS/10% goat serum for 1 h. Cells were washed three more times and affixed to slides with Fluoroshield histology mounting medium (Merck). The entire staining procedure was performed at room temperature. Cell were viewed on an IX83 inverted microscope (Olympus, Shinjuku, Japan) and images taken using an Orca R2 digital CCD camera (Hamamatsu Photonics, Hamamatsu, Japan) and cellSens Dimension 1.18 software (Olympus).

All qualitative experiments of this type were performed three times independently and used if the findings were consistent. If one of the three experiments appeared to produce contrasting results, then two additional independent tests were performed. If four of the five experiments showed the same result, then the outlier was assumed to be due to technical issues and discounted. If results were still inconsistent, then the variability of results is reported in the text, and examples of both results are shown.

For quantified analysis of protein (co-)aggregation, tubes containing plasmids were coded and randomized. The researcher who transfected these into cells, and then analyzed the proportion of cells with (co-)aggregation, was therefore blinded as to which plasmid(s) each cell was expressing. Images of the first 10 transfected cells per coverslip were taken (or images of all cells, if 10 could not be found) and then analyzed once all images were collected. Analysis was performed in ImageJ (NIH). For the purposes of quantification, an aggregate was defined as any compact area of intense signal more than 1 μm in diameter. Similarly, a co-aggregate was defined as any compact area of intense signal more than 1 μm in diameter that was visible in two channels. Only after quantification were the samples decoded for statistical analysis using GraphPad Prism.

## 3. Results

### 3.1. Presence of Multiple Insoluble Proteins in Individual Brain Samples

In order to investigate possible co-aggregation or parallel aggregation of proteins implicated in mental illness, a cohort of insular cortex samples was collected, consisting of victims of suicide (*n* = 15), control individuals (*n* = 11) and smaller numbers of patients with MDD (*n* = 4) or Alzheimer’s disease (*n* = 3). The insular cortex was used because of its previous association with neurological and psychiatric disorders [24].

**Figure 2:**
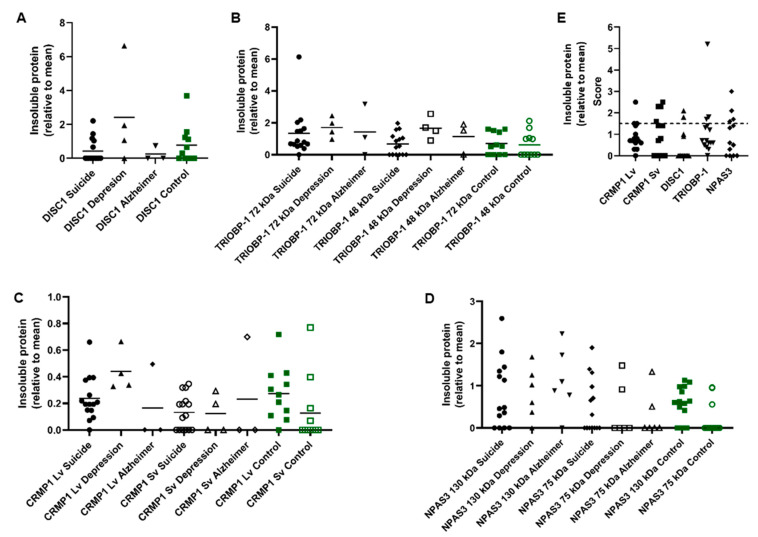
Insoluble protein in human insular cortex samples, from 15 victims of suicide, 11 control individuals, 4 patients with MDD and 3 patients with Alzheimer’s disease. (**A**–**D**) Quantified levels of DISC1 (**A**), TRIOBP-1 (**B**), CRMP1 (**C**) and NPAS3 (**D**) protein seen in the insoluble protein fraction of post mortem insular cortex samples. Values are normalized to a common sample loaded on each membrane. Original Western blot data for DISC1, TRIOBP-1 and CRMP1 are in Figure S1. NPAS3 data (**D**) have been published previously [13] but are reanalyzed and summarized here for comparison. The NPAS3 data also include a few additional samples, which were not available for this study. (**E**) For the protein isoforms analyzed further (CRMP1 Lv and Sv, DISC1 70 kDa, TRIOBP-1 72 kDa, NPAS3 130 kDa), the proteins present in the insoluble pellet at a level at least 50% higher than the mean, which are interpreted as aggregating for the purposes of this study. The number of proteins found to be insoluble (aggregating) in each sample can be seen in Figure 3. Graphs prepared using GraphPad Prism.

These samples were homogenized and the insoluble protein fractions of each sample were then purified. These fractions were investigated by Western blotting to determine if insoluble (aggregating) CRMP1, DISC1 and/or TRIOBP-1 was present in them (Figure 2A–D and Figure S1). The original, non-purified brain homogenates were also Western blotted for comparison (Figure S2). Some level of insoluble protein was seen in many of the samples, with a few instances of individuals showing very high amounts of one specific insoluble protein. Notably, one MDD patient showed very high levels of insoluble DISC1 aggregation (Figure 2A and Figure S1B) and one suicide victim similarly showed high levels of insoluble TRIOBP-1 (Figure 2B and Figure S1A, major 72 kDa species), while individuals of various diagnoses expressed high levels of CRMP1 (both the long variant, Lv, and short variant, Sv) as insoluble proteins (Figure 2C and Figure S1C, Lv: 70 kDa, Sv: 65 kDa). NPAS3 has previously been analyzed in these samples [13], with some individuals showing high levels of insoluble NPAS3 (Figure 2D, major 120 kDa species).

**Figure 3:**
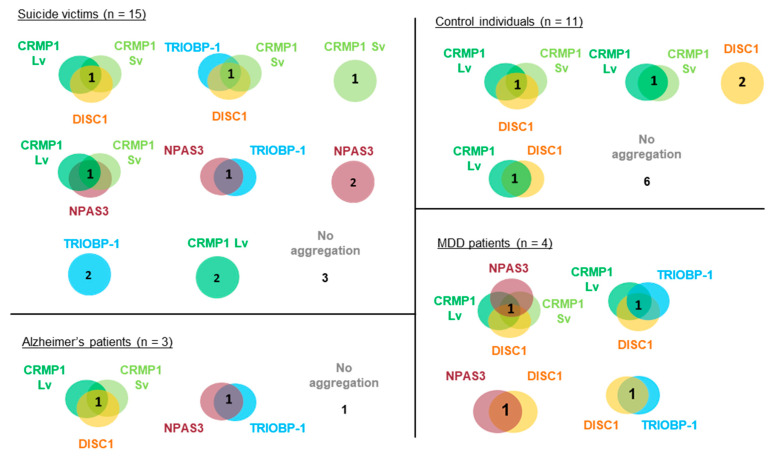
Detailed breakdown of co-occurrence of insoluble proteins among the insular cortex samples. Each circle or Venn diagram represents one or more individuals (as indicated by the number) and the proteins that are insoluble in the insular cortex sample from that individual. Insoluble is defined as a level of a specific protein in the insoluble fraction that is at least 1.5× the average across all 33 samples tested. DISC1, NPAS3 and TRIOBP-1 represent the major 70 kDa, 130 kDa and 72 kDa species, respectively.

In the majority of cases, levels of insoluble protein were not normally distributed, with many individuals showing little or none of a specific protein, and others showing considerably higher levels. For the purposes of this experiment, we considered any sample that contained more than 1.5× the mean level of an individual protein to potentially contain that protein in an aggregating state (Figure 2E). Nine of the 15 suicide victims contained at least one potential aggregating protein by this definition (60%), compared to 5 out of 11 control individuals (45%, Figure 2E). The 4 MDD and 3 Alzheimer’s samples all contained potential aggregating proteins, according to this definition. Of the samples showing aggregation, more than half showed at least two proteins to be potentially aggregating, with some showing three and one MDD patient having four (Figure 3). It therefore seems likely that multiple proteins do aggregate in subsets of patients. The proportion of individuals expressing multiple aggregating proteins suggests that these are more likely to be a result of each protein aggregating individually (parallel aggregation), rather than through the active effect of one aggregating protein on the aggregation state of another (co-aggregation). The proteins that were most commonly found to be insoluble together in samples were CRMP1 Lv, CRMP1 Sv and DISC1; however, incidences of insoluble NPAS3 and TRIOBP-1 being present were also seen (Figure 3). There was no obvious correlation between levels of insolubility of any two individual proteins (Figure S3).

### 3.2. Pairwise Co-Expression Studies in Neuroblastoma Cells Show only DISC1 Readily Forms Co-Aggregates, with Both CRMP1 and TRIOBP-1

**Figure 4:**
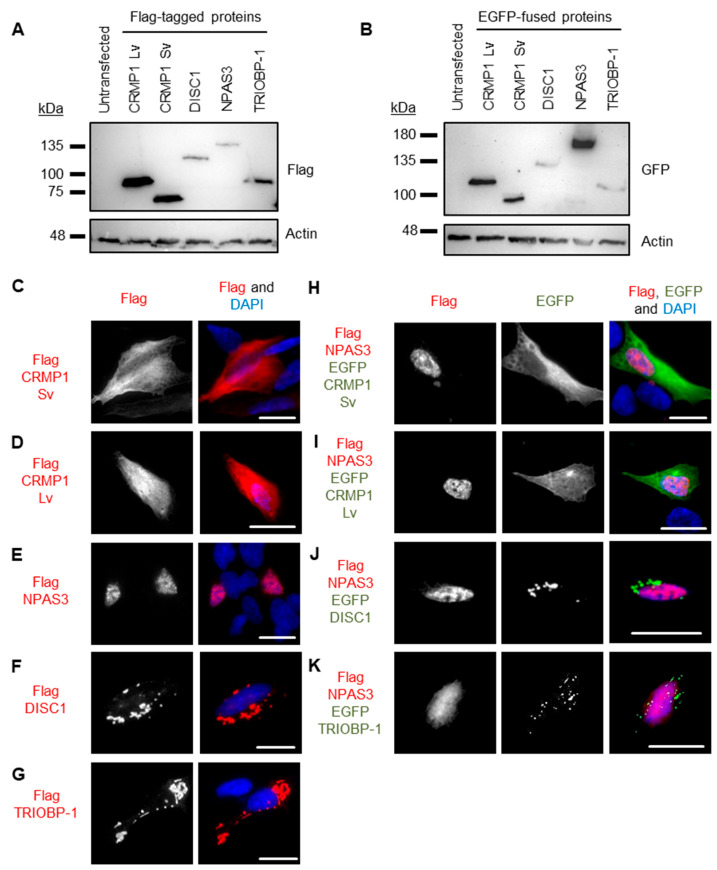
Systematic pairwise testing of co-aggregation in cell culture. (**A**) Western blots of Flag-tagged proteins used in these experiments, expressed in HEK293 cells. (**B**) Equivalent blot of EGFP-fused proteins used in this experiment. Remaining images show constructs expressed in SH-SY5Y neuroblastoma cells: (**C**) Flag-tagged CRMP1 Sv, not aggregating. (**D**) Flag-tagged CRMP1 Lv, not aggregating. (**E**) Flag-tagged NPAS3, not aggregating. (**F**) Flag-tagged DISC1, aggregating. (**G**) Flag-tagged TRIOBP-1, aggregating. (**H**) Flag-tagged NPAS3 and EGFP-fused CRMP1 Sv, neither aggregating. (**I**) Flag-tagged NPAS3 and EGFP-fused CRMP1 Lv, neither aggregating. (**J**) Flag-tagged NPAS3 and EGFP-fused DISC1, only DISC1 is aggregating. (**K**) Flag-tagged NPAS3 and EGFP-fused TRIOBP-1, only TRIOBP-1 is aggregating. All cell photos are typical of 3 or more independent experiments. Scale bars represent 10 μm. Figure S5 shows versions of the experiments in (**H**) to (**K**) using the reciprocal vectors.

Genes coding for CRMP1 (Lv and Sv), DISC1 (L isoform), NPAS3 and TRIOBP-1 were each expressed in two plasmid vectors, one that added a Flag tag to them and one that fused them to EGFP. Protein expression was confirmed by Western blotting (Figure 4A,B). These were then expressed in SH-SY5Y neuroblastoma cells to establish whether or not they spontaneously formed visible aggregates. While not a perfect model of human neurons, these cells were selected as they provide a good balance between having neuronal characteristics, while still being practical for transfection with many different plasmid vectors. Using Flag-tagged proteins, CRMP1 was found in the cell body (Lv and Sv, Figure 4C,D) and NPAS3 was primarily found in the nucleus (Figure 4E, with occasional cells showing cytoplasmic localization instead). In contrast, DISC1 and TRIOBP-1 were each consistently seen to aggregate in the cell body (Figure 4F,G), by which we mean that they were seen to form intense compact accumulations in the body of the cell that give us visibly more signal than any diffuse staining in the cell body. Such structures correspond well to protein insolubility in the cell, reinforcing that they represent aggregates [9,10,13,19]. These expression patterns match those seen in previous work, in which DISC1 and TRIOBP-1 aggregated readily when expressed in cells [7,9,10,19], while CRMP1 Sv and NPAS3 did so only rarely, or when an additional factor was involved, such as oxidative stress, mutation or co-expression with another protein [9,12,13]. There were no changes in expression pattern of any of the Flag-tagged proteins when they were co-expressed with EGFP (Figure S4A–F). EGFP-fused versions of these proteins behaved identically to their Flag-tagged counterparts (Figure S4G–M) with the exception of EGFP-CRMP1, which was sometimes seen to form aggregate-like structures (a minority of cells expressing EGFP-CRMP1 Sv, a majority expressing EGFP-CRMP1 Lv, Figure S4H,I).

**Figure 5:**
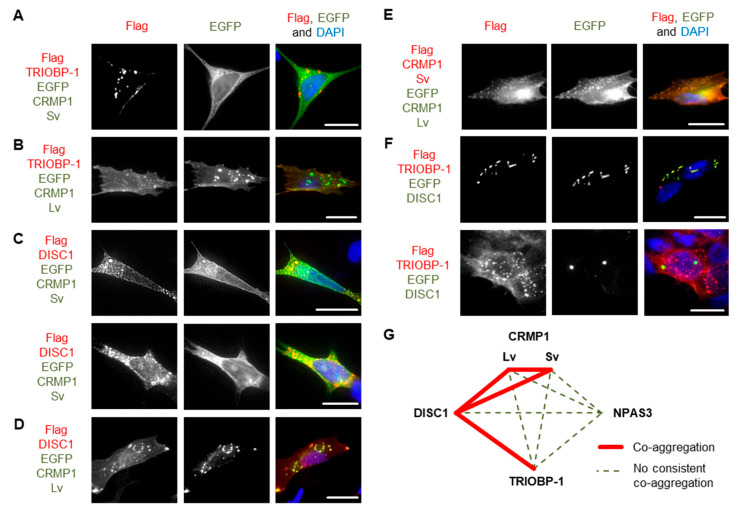
Further systematic pairwise testing of co-aggregation in cell culture. Images show constructs co-expressed in SH-SY5Y neuroblastoma cells: (**A**) Flag-tagged TRIOBP-1 and EGFP-fused CRMP-1 Sv, only TRIOBP-1 aggregates. (**B**) Flag-tagged TRIOBP-1 and EGFP-fused CRMP-1 Lv, only TRIOBP-1 aggregates. (**C**) Flag-tagged DISC1 and EGFP-fused CRMP-1 Sv, example of cells, one with co-aggregation and one with aggregation of DISC1. (**D**) Flag-tagged DISC1 and EGFP-fused CRMP-1 Lv showing co-aggregation. (**E**) Flag-tagged CRMP1 Sv and EGFP-fused CRMP1 Lv showing co-aggregation. (**F**) Flag-tagged TRIOBP-1 and EGFP-fused DISC1, example of cells with co-aggregation and parallel aggregation. (**G**) Summary of co-aggregation results. All cell photos are typical of three or more independent experiments. Scale bars represent 10 μm. Figure S6 shows versions of these experiments using the reciprocal vectors.

Pairs of proteins were then systematically co-expressed in SH-SY5Y to determine whether they could co-aggregate in these cells. In all cases, experiments were performed with one protein being Flag-tagged and the other fused to EGFP, and then a reciprocal experiment was performed to verify the results with the other combination of vectors. Unless otherwise noted, all results were consistent, regardless of which protein had the Flag tag and which was fused to EGFP. NPAS3 showed no consistent signs of co-aggregation with any of the other proteins (Figure 4H–K and Figure S5). Similarly, while TRIOBP-1 formed aggregates in cells containing CRMP1 Sv, there was no sign of co-aggregation (Figure 5A and Figure S6A). Co-aggregation of TRIOBP-1 and CRMP1 Lv was seen in some cells, but only when TRIOBP-1 was Flag-tagged and CRMP1 fused to EGFP, not in the reverse situation (Figure 5B and Figure S6B). In contrast, DISC1 was seen to co-aggregate with both variants of CRMP1 in many, but not all, cells with Sv and in most cells with Lv (Figure 5C,D and Figure S6C–E). Subpopulations of CRMP1 Lv and Sv also co-aggregated with each other in a majority of cells examined (Figure 5E and Figure S6F). Notably, however, while both DISC1 and TRIOBP-1 were seen to each aggregate in almost all cells in which they were co-expressed, in some cells they were seen to clearly co-aggregate with each other, while in others they aggregated in parallel (Figure 5F and Figure S6G). This demonstrates that each of DISC1 and TRIOBP-1 can aggregate independently of each other in a single cell (in agreement with the fact that each can aggregate when expressed alone), although the existence of cells with co-aggregation also demonstrates them to be capable of direct interaction when in an aggregated state.

These results confirm one previous report of co-aggregation from the literature, that of DISC1 with CRMP1 Sv [7], and indicate two novel ones: DISC1 with TRIOBP-1 and DISC1 with CRMP1 Lv (Figure 5G). All other pairs of proteins examined were not seen to co-aggregate, or only appeared to do so in a very small minority of cells (<5%).

### 3.3. No Obvious Effect of CRMP1 on the Extent of DISC1 Aggregation

**Figure 6:**
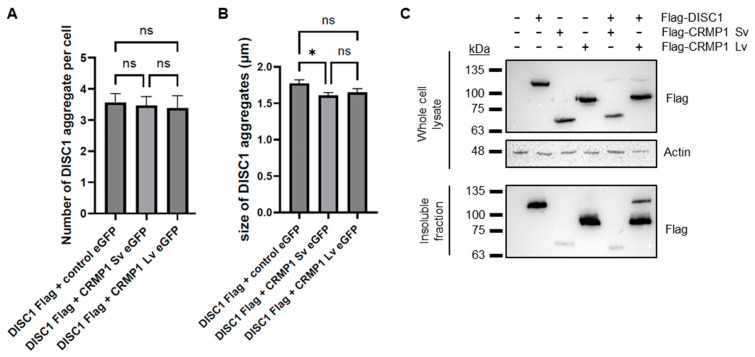
Investigating the co-aggregation of DISC1 and CRMP1. (**A**,**B**) Co-expression of Flag-DISC1 with EGFP, EGFP-CRMP1 Sv or EGFP-CRMP1 Lv in SH-SY5Y cells, analyzed by immunofluorescent microscopy in a blinded, quantified manner. For the purpose of this assay, an aggregate was defined as a punctate structure at least 1 µm in diameter. Results are an average of 10 coverslips per plasmid combination (9 in the case of DISC1 + EGFP), with 10 transfected cells examined per coverslip (or as many transfected cells as could be found). (**A**) Mean number of Flag (DISC1) aggregates per cell. (**B**) Mean size of DISC1 aggregates in cells displaying DISC1 aggregates. *: *p* < 0.05, ns: not significant, according to one-way ANOVA. (**C**) Western blots from an insoluble fraction purification assay. HEK293 cells were transfected with various combinations of Flag-tagged DISC1, CRMP1 Sv and Lv, lysed and then had their insoluble protein fraction purified. Samples are shown from both the unfractionated cell lysate and the purified insoluble fraction, and are representative of three independent experiments.

A previous report demonstrated that CRMP1 co-aggregates with huntingtin (HTT) in Huntington’s disease, but in doing so reduces the extent of aggregation and neurotoxicity of huntingtin [25]. It is therefore plausible that it has a similar effect on DISC1. To test this, we performed a quantitative assay in which Flag-tagged DISC1 was co-transfected into SH-SY5Y cells with EGFP alone, EGFP-CRMP1 Sv or EGFP-CRMP1 Lv, by a researcher who was blinded as to which plasmid was in each set of cells. This researcher then assessed aggregation, before having the data decoded. Co-expression with EGFP-CRMP1 Sv or Lv had no effect on the number of DISC1 aggregates per cell compared to co-expression with EGFP alone (Figure 6A), although the average size of DISC1 aggregates co-expressed with CRMP1 Sv was slightly smaller (1.415 ± 0.039 μm when co-expressed with EGFP-CRMP1 Sv, compared to 1.552 ± 0.051 μm when co-expressed with EGFP alone, Figure 6B). There was no significant difference in the number or size of co-aggregates of DISC1 with CRMP1 Sv or Lv (Figure S7A–C), although levels of co-aggregates of each were higher than was seen with EGFP alone. There was also no significant difference in the number of aggregates of CRMP1 Sv compared to Lv (Figure S7D). CRMP1 therefore seems to have a minimal effect on DISC1 aggregation, at least in this assay.

As an alternative approach, DISC1, CRMP1 Sv and/or CRMP1 Lv were also transfected into HEK293 cells, which were subsequently lysed (all constructs were confirmed to show the same expression pattern in HEK293 as in SH-SY5Y, Figure S8). These cell lysates then had their insoluble protein fraction purified, in a method directly analogous to that used on the brain samples. DISC1 and CRMP1 Lv were both prominent in the insoluble protein fraction, while CRMP1 Sv was present at a much lower level, indicating that CRMP1 Sv may have a lower aggregation propensity than DISC1 or CRMP1 Lv (Figure 6C). There was no clear effect of CRMP1 on levels of insoluble DISC1.

### 3.4. DISC1 Can Induce Aggregation of TRIOBP-1, with No Individual Domain of TRIOBP-1 Sufficient for This

**Figure 7:**
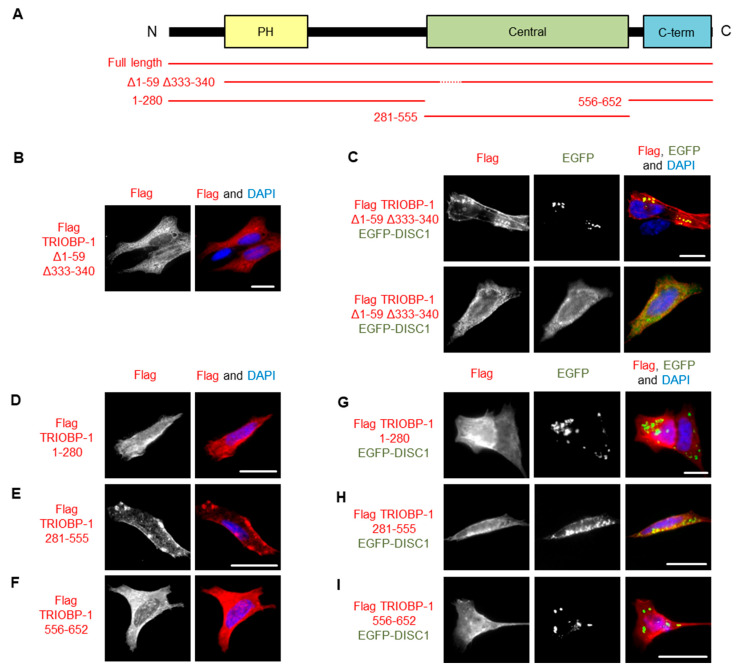
Investigating the co-aggregation of DISC1 and TRIOBP-1 in SH-SY5Y cells. (**A**) Schematic of the major domains of TRIOBP-1 (Pleckstrin homology domain, central coiled-coiled domain and C-terminal coiled-coil domain) and locations included in the plasmid vectors used here. (**B**) Expression pattern of TRIOBP-1 Δ1–59 Δ333–340, which does not aggregate by itself. (**C**) When this construct is co-expressed with DISC1, it co-aggregates with DISC1 in some cells but not in others. One example of each is shown. (**D**) The N-terminal section of TRIOBP-1, including the PH domain, does not aggregate alone. (**E**) The central coiled-coil domain of TRIOBP-1 aggregates when expressed alone. (**F**) The C-terminal coiled-coil domain of TRIOBP-1 does not aggregate when expressed alone. (**G**) The N-terminal sections of TRIOBP-1 do not co-aggregate with DISC1. (**H**) The central coiled coil-domain of TRIOBP-1 shows only limited co-aggregation with DISC1. (**I**) The C-terminal coiled coil-domain of TRIOBP-1 does not co-aggregate with DISC1. All cell photos are typical of three or more independent experiments. Scale bars represent 10 μm.

To further investigate the co-aggregation of DISC1 and TRIOBP-1, we utilized our recently developed TRIOBP-1 mutant, Δ1–59Δ333–340, which lacks its optionally translated N-terminus and a short loop region in the middle of the protein (Figure 7A and Figure S9A), abolishing its ability to aggregate [6]. We therefore expressed both this truncated form of TRIOBP-1 and full length TRIOBP-1, both labelled with Flag tags. These were each co-expressed with full length DISC1 fused to EGFP. Notably, while the TRIOBP-1 mutant did not aggregate when expressed alone or with EGFP (supp. Figure 7B), it did co-aggregate with full length DISC1 in some cells (Figure 7C), indicating that DISC1 can induce aggregation of TRIOBP-1. In other cells, DISC1 and the TRIOBP-1 mutant still colocalized, but without signs of aggregation (Figure 7C).

To investigate the co-aggregation further, DISC1 was co-transfected with fragments of TRIOBP-1 representing its disordered N-terminus and PH domain, its central coiled-coil domain or its C-terminal coiled-coil domain. In agreement with previous results [19], of these, only the central coiled-coil domain forms aggregates when expressed alone (Figure 7D–F and Figure S9B). When co-expressed with DISC1, neither the N-terminal region nor the C-terminal coiled-coil domain showed signs of co-aggregation, while the central coiled-coil domain showed only weak indications compared to the full length protein, either alone or when expressed together (Figure 7G–I). It therefore appears that DISC1 needs to interact with multiple sections of the TRIOBP-1 protein in order to induce co-aggregation.

## 4. Discussion

Impaired proteostasis, and the accumulation of insoluble protein, is an established and well recognized feature of neurodegenerative diseases. Some diseases are characterized by a single aggregating protein, such as huntingtin in Huntington’s disease, while others are associated with a variety of distinct proteinopathies, such as in amyloid lateral sclerosis or frontotemporal lobe dementia. The reasons that these proteins begin to aggregate vary but include both inherited and spontaneous mutations in the genes encoding them, as well as environmental stresses. Recently, various proteins have been implicated as forming aggregates in major mental illnesses, although the relationship between these proteins remains unclear.

One such protein, DISC1 has been found to aggregate in the same brain samples as two other proteins implicated in mental illness, CRMP1 and dysbindin-1 [7,9], as well as two proteins related to neurodegenerative disease, huntingtin and TDP-43 [26,27]. In all cases, DISC1 was found to co-aggregate directly with this other protein in cell culture or other in vitro assays. It therefore could reasonably be predicted that the co-occurrence of aggregating (insoluble) proteins in the brains of patients with mental illness would typically be the result of direct co-aggregation of the proteins. In our data, however, while instances of multiple proteins aggregating in a single brain sample were relatively common, they are not high enough to imply that one protein frequently affects the aggregation state of another. Partially consistent with this is the fact that, of the four proteins we investigated here, CRMP1, DISC1, NPAS3 and TRIOBP-1, only two pairs show direct co-aggregation. One of these is the previously reported CRMP1-DISC1 aggregation and the other is a novel pair: DISC1 and TRIOBP-1. We did not study dysbindin-1 here, as we were unable to get consistent antibody staining against it in our brain homogenate samples.

Co-aggregation was studied by pairwise expression of proteins in SH-SY5Y cells, one with a Flag tag and one with an EGFP fusion protein for detection. In most instances, changing which protein was in each vector did not have an effect on the results, although it was notable that CRMP1 appeared to show increased aggregation when fused to EGFP. This is potentially because the relatively large size (27 kDa) of the fusion protein increases the stability of the protein, meaning that if the protein began to misfold, it would be more likely to survive as an aggregating protein, as opposed to being destroyed by the proteasome. As a precaution, however, all pairs of proteins were expressed with both combinations of protein and plasmid vector to minimize any effect of the EGFP fusion protein.

The ability of DISC1 and CRMP1 Sv to co-aggregate was reported previously, with the two being insoluble in the same brain samples and also aggregating in cell lines [7]. In that previous study, aggregation of CRMP1 Sv was only seen when co-expressed with DISC1 and appeared more prominent than in our experiments [7]; however, this may be because those authors used fluorescent tags on both proteins, which can lead to heightened aggregation of CRMP1, as seen here. This study also found GFP-tagged CRMP1 Lv to aggregate [7], which matches our results, although our data showed it not to consistently aggregate without this fluorescent fusion protein. Co-aggregation of CRMP1 Lv and DISC1 had not been studied before, to our knowledge, nor has the co-aggregation of the two CRMP1 isoforms with each other, with our insolubility assays suggesting that CRMP1 Lv may be more prone to insolubility than CRMP1 Sv. This was not seen in the quantitative immunofluorescence assay, which instead looks at aggregate distribution, rather than the quantity of aggregating protein molecules.

TRIOBP-1 was first implicated as aggregating in schizophrenia using a variation of the same process used to detect insoluble CRMP1 [10], and was later validated in a distinct set of samples from schizophrenia and MDD patients [6]. Aggregation of TRIOBP-1 was therefore identified separately from that of DISC1, and no interaction between the two proteins has been previously reported, to the best of our knowledge. However, they both share a few common features, including expression in the brain, roles in the actin cytoskeleton [28,29,30] and at least two mutual protein interaction partners in NDEL1 [31,32,33,34,35] and TRIO [28,36]. While DISC1 could co-aggregate with full length TRIOBP-1, it could not clearly do so with individually expressed domains of TRIOBP-1, indicating that multiple points of contact between the two proteins are likely required for co-aggregation to occur. It is interesting to note that not all cells showed co-aggregation of DISC1 with TRIOBP-1, with parallel aggregation of the two sometimes seen in the same cell. While it is not a surprise that each protein is capable of aggregating independently of each other, this does imply that aggregates of one protein (DISC1 or TRIOBP-1) may preferentially recruit other molecules of the same protein to aggregate over recruiting molecules of the other protein. The reason why some cells therefore show parallel aggregation and others show co-aggregation is therefore unclear; however, one possibility is that it is defined by the initial misfolded molecules that “seed” the aggregation. If these initial molecules are of only one protein, then an aggregate principally of that protein may form, while if the initial molecules are a complex of DISC1 and TRIOBP-1, then a co-aggregate forms instead. It is currently unknown if DISC1 and TRIOBP-1 interact when both are in a folded functional state; however, the nature of DISC1 as a scaffold protein [37] makes it more likely.

It is also notable, however, that DISC1 could also co-aggregate with a mutant form of TRIOBP-1 that lacked its two aggregation-critical regions, indicating that co-aggregation of the two proteins can be driven by the aggregation propensity of DISC1 alone, and that this is a distinct mechanism from that by which TRIOBP-1 aggregates alone. That some cells instead showed this non-aggregating form of TRIOBP-1 and DISC1 to colocalize, but not co-aggregate, further implies that the proteins may interact in a native state, with the non-aggregating TRIOBP-1 able to act as a stabilization factor to the aggregation-prone DISC1.

The main limitation of the post mortem brain tissue work is that the comparatively low levels of aggregation seen in samples from suicide victims may behave differently to higher levels seen previously in schizophrenia. Similarly, for the cell culture-based work, we are examining over-expressed protein in immortalized cells; therefore, it is possible that its aggregation may differ from that of endogenous protein in the brain. That the two lines of research yield a similar picture, however, supports their use as a proof of concept that a combination of co-aggregation and parallel aggregation of mental illness-related proteins being present in the brain.

While research into proteinopathy in major mental illness is still at a relatively early stage, the fact that individual proteins aggregate in subsets of patients raises the exciting possibility that these aggregates may characterize specific biological subgroups of these conditions. With greater understanding of this phenomenon, it is plausible that detection of these protein aggregates, or biological correlates of them outside of the brain, could ultimately aid in biological diagnosis and/or selection of personal therapeutic approaches. Understanding the relationship between aggregation of the various proteins would therefore be critical to such an approach. Beyond that, the fact that we do not see clear correlation between aggregation of the various proteins in patients, with parallel aggregation in some patients but not others, supports the idea that aggregation of specific proteins is relevant to illness, as opposed to the pathology of these conditions instead being associated with increased levels of insoluble/aggregating proteins generally.

## 5. Conclusions

Based on this study, and consistent with previous data, it seems that multiple proteins can aggregate in the brain of the same individual with mental illness; however, in the majority of cases this is because each protein aggregates independently of each other (parallel aggregation). Instances of the aggregation of one protein having a direct effect on the aggregation of another (co-aggregation) are seemingly less common, and likely limited to specific pairs of proteins, with DISC1, in particular, able to induce aggregation of multiple proteins.

## Data Availability

All principal data is included in this manuscript or the supplementary material. For further information on data replicates or similar, please contact the authors.

## Supplementary Materials

The following supporting information can be downloaded at: https://www.mdpi.com/article/10.3390/cells12141848/s1, Figure S1: Western blots of insoluble protein fractions, purified from human insular cortex samples; Figure S2: Western blots of non-purified insular cortex sample homogenates; Figure S3: Pairwise comparisons of insoluble protein levels in the insular cortex samples; Figure S4: Expression patterns of Flag-tagged proteins expressed with EGFP alone and of EGFP-fused proteins; Figure S5: Versions of experiments in Figure 4H–K using reciprocal plasmid vectors; Figure S6: Versions of experiments in Figure 5A–G using reciprocal plasmid vectors; Figure S7: Quantified data on CRMP1 and DISC1 co-aggregation; Figure S8: Expression patterns of proteins in HEK293 match those in SH-SY5Y; Figure S9: Western blots showing expression of additional TRIOBP-1 constructs used in this study; Table S1: Sources and generation of plasmid vectors used in this study; Table S2: Primers used for cloning in this study. Appendix S1: Amino acid sequences of proteins expressed from plasmid vectors.
