# Supplementary material for: Co-Aggregation and Parallel Aggregation of Specific Proteins in Major Mental Illness
Source: Cells. 2023 Jul 13;12(14):1848. doi: 10.3390/cells12141848 (PMC10378145; doi:10.3390/cells12141848)
Supplement: Supplementary file 1 [file cells-12-01848-s001.zip › cells-2488896-supplementary.pdf]

Co-aggregation and parallel aggregation of specific proteins in major mental illness

Bobana Samardžija*, Maja Juković*, Beti Zaharija, Éva Renner,

Miklós Palkovits & Nicholas J. Bradshaw

Supplementary information

Table S1:	Sources and generation of plasmid vectors used in this study
Table S2:	Primers used for cloning in this study
Figure S1:	Western blots of insoluble protein fractions, purified from human insular cortex samples
Figure S2:	Western blots of non-purified insular cortex sample homogenates
Figure S3:	Pairwise comparisons of insoluble protein levels in the insular cortex samples
Figure S4:	Expression patterns of Flag-tagged proteins expressed with EGFP alone and of EGFP-fused proteins
Figure S5:	Versions of experiments in figure 4H-K using reciprocal plasmid vectors
Figure S6:	Versions of experiments in figure 5A-G using reciprocal plasmid vectors
Figure S7:	Quantified data on CRMP1 and DISC1 co-aggregation
Figure S8:	Expression patterns of proteins in HEK293 match those in SH-SY5Y
Figure S9:	Western blots of additional TRIOBP-1 vectors
Appendix S1:	Amino acid of proteins expressed using plasmid vectors in this study

No.	Vector backbone	Gene insert	Origin
1	pENTR1A no ccDB	(None)	Campeau et al (2009) PLOS One 4:e6529 (Addgene, clone 17398)
2	pdcDNA-FlagMyc	(None)	BCCM/LMBP Plasmid Collection, clone LMBP 4705
3	pDEST-CMV-EGFP	(None)	Agrotis et al (2019) Autophagy 15:976-997 (Addgene, clone 123215)
4	pcDNA3.1	CRMP1 Sv	Bader et al. (2012) Hum. Mol. Genet. 21:4406-4418 (gift from Carsten Korth)
5	pcDNA3.1	CRMP1 Lv	Bader et al. (2012) Hum. Mol. Genet. 21:4406-4418 (gift from Carsten Korth)
6	pENTR1A	CRMP1 Sv	Subcloned from plasmid 4 in two rounds of PCR, with primers A & C, then D & E. Restriction digested and ligated into the *KpnI* and *XbaI* sites of plasmid 1.
7	pENTR1A	CRMP1 Lv	Subcloned from plasmid 5 in two rounds of PCR, with primers B & C, then D & E. Restriction digested and ligated into the *KpnI* and *XbaI* sites of plasmid 1.
8	pENTR223	DISC1	DNASU Plasmid Repository, clone HsCD00516321
9	pENTR223.1	NPAS3	DNASU Plasmid Repository, clone HsCD00080332
10	pENTR1A	TRIOBP-1	Bradshaw et al (2017) J. Biol. Chem. 292:9583-9598 (gift from Carsten Korth)
11	pdcDNA-Flag	CRMP1 Sv	LR clonase recombination of plasmids 2 and 6
12	pdcDNA-Flag	CRMP1 Lv	LR clonase recombination of plasmids 2 and 7
13	pdcDNA-Flag	DISC1	LR clonase recombination of plasmids 2 and 8
14	pdcDNA-Flag	NPAS3	Samardžija et al (2021) J. Pers. Med. 11:1070
15	pdcDNA-Flag	TRIOBP-1	Bradshaw et al (2017) J. Biol. Chem. 292:9583-9598 (gift from Carsten Korth)
16	pdcDNA-Flag	TRIOBP-1 Δ1-59Δ333-340	Zaharija et al (2022) Int. J. Mol. Sci. 23:11048
17	pdcDNA-Flag	TRIOBP-1 1-280	Bradshaw et al (2017) J. Biol. Chem. 292:9583-9598 (gift from Carsten Korth)
18	pdcDNA-Flag	TRIOBP-1 281-555	Bradshaw et al (2017) J. Biol. Chem. 292:9583-9598 (gift from Carsten Korth)
19	pdcDNA-Flag	TRIOBP-1 556-652	Bradshaw et al (2017) J. Biol. Chem. 292:9583-9598 (gift from Carsten Korth)
20	pDEST-CMV-N-EGFP	(Empty control)	LR clonase recombination of plasmids 1 and 4
21	pDEST-CMV-N-EGFP	CRMP1 Sv	LR clonase recombination of plasmids 4 and 6
22	pDEST-CMV-N-EGFP	CRMP1 Lv	LR clonase recombination of plasmids 4 and 7
23	pDEST-CMV-N-EGFP	DISC1	LR clonase recombination of plasmids 4 and 8
24	pDEST-CMV-N-EGFP	NPAS3	LR clonase recombination of plasmids 4 and 9
25	pDEST-CMV-N-EGFP	TRIOBP-1	LR clonase recombination of plasmids 4 and 10

Table S1. Sources and generation of plasmid vectors used in this study. See table S2 for references to primers.

Label	Name	Primer
A	CRMP1-1sv-salF	GAAGTCGACATGTCGTACCAG
B	CRMP1-1lv-salF	GTAAGTCGACATGGCGGACCG
C	CRMP1-572sv-ecoR	GTAGAATTCTCATCAACCGAGGCTG
D	Extension F	GCTATAAGGATCCGGTACCTAGTCGACATG
E	Extension R	GGCACCAGCTCGAGTCTAGAATTCTCATC

Table S2. Primers used for cloning in this study. “Label” corresponds to how they are referred to in table S1.

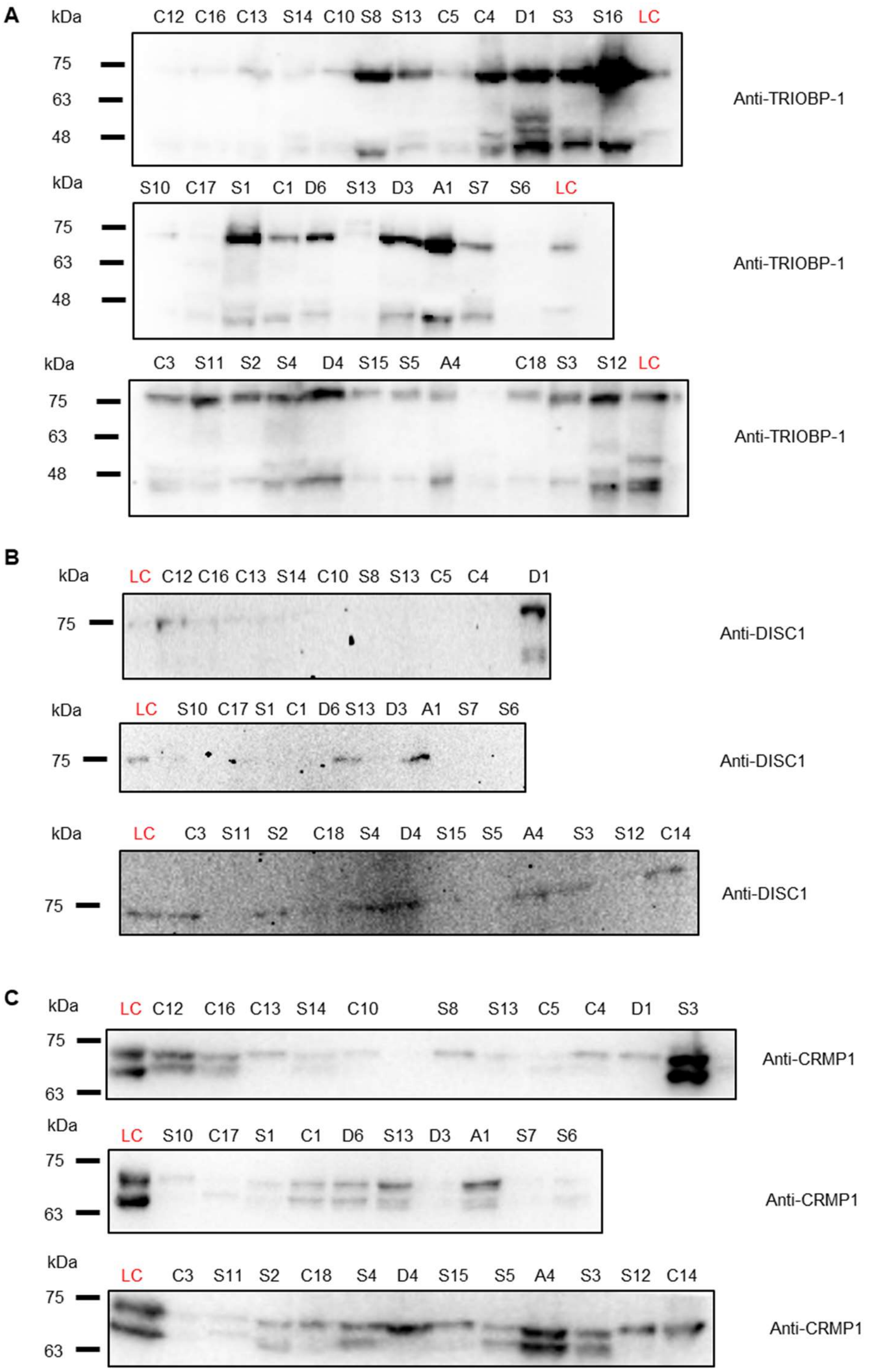

Figure S1. Western blots of the insoluble protein fractions, purified from insular cortex samples. Samples are labelled with diagnostic status (S: victim of suicide, C: control individual, D: major depressive disorder patient, A: Alzheimer's disease patient) and a unique number to allow comparison between blots in figures S1 and S2, which displays the corresponding non-purified homogenates. LC is a loading control: a standard sample loaded on all membranes to allow normalisation of signal quantification between gels.

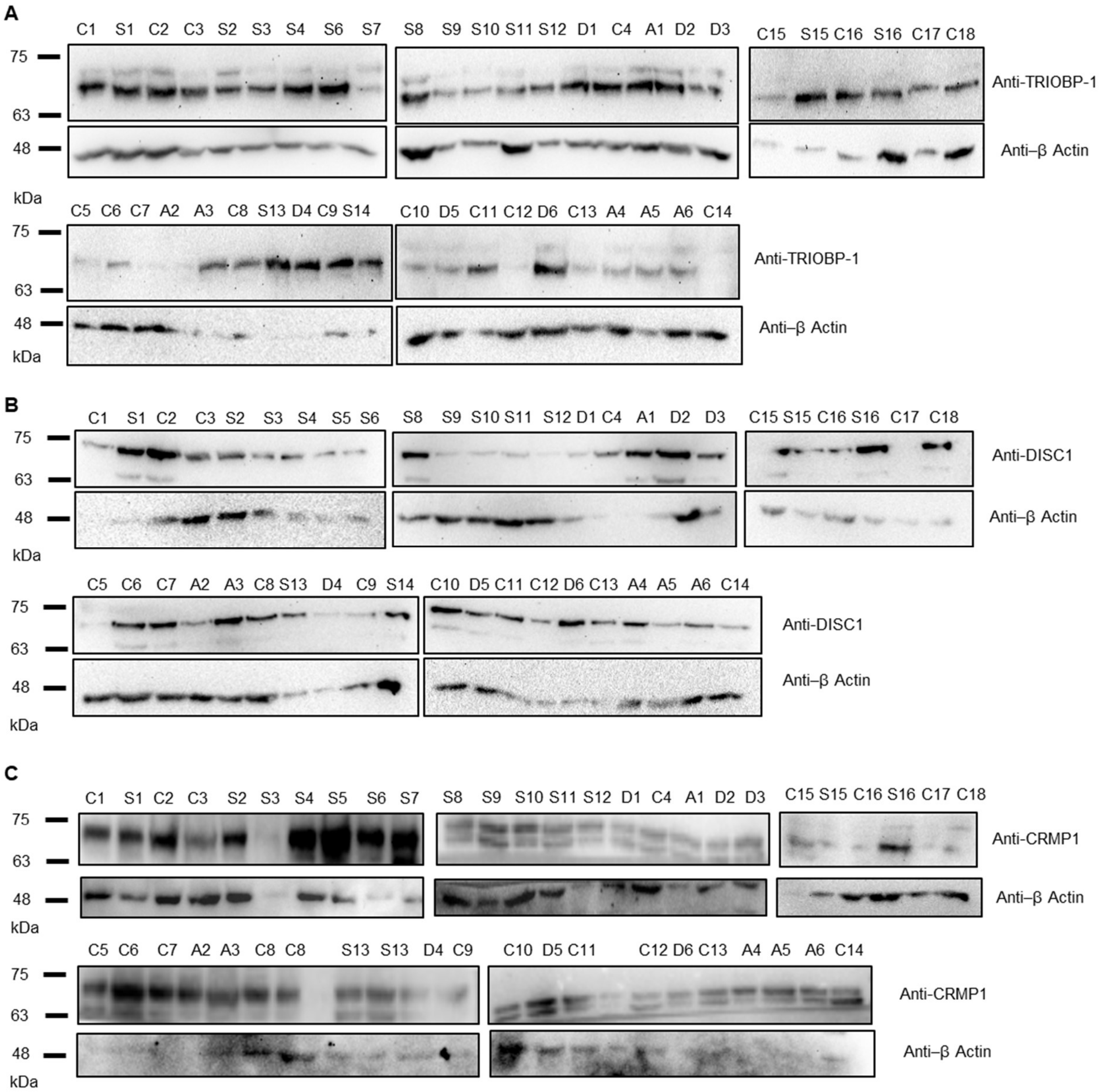

Figure S2. Western blots of insular cortex sample homogenates. Samples are labelled with diagnostic status (S: victim of suicide, C: control individual, D: major depressive disorder patient, A: Alzheimer's disease patient) and a unique number to allow comparison between blots in figures S1 and S2, which displays insoluble protein fractions, purified from these homogenates.

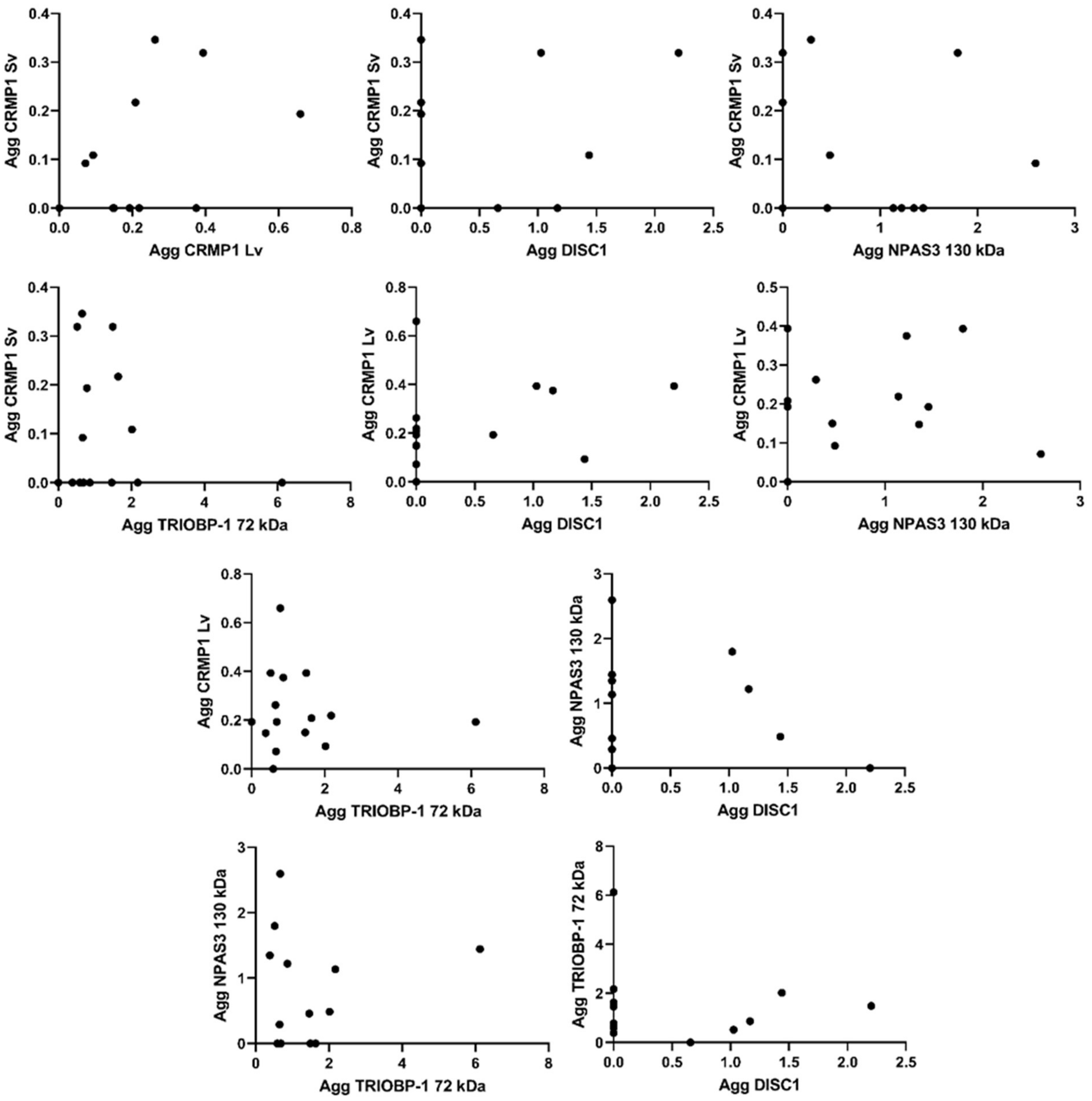

Figure S3. Comparison of levels of CRMP1 sv, CRMP1 lv, DISC1 (75kDa), NPAS3 (130kDa) and TRIOBP-1 in individuals. Based on data shown in figures 2 and S1.

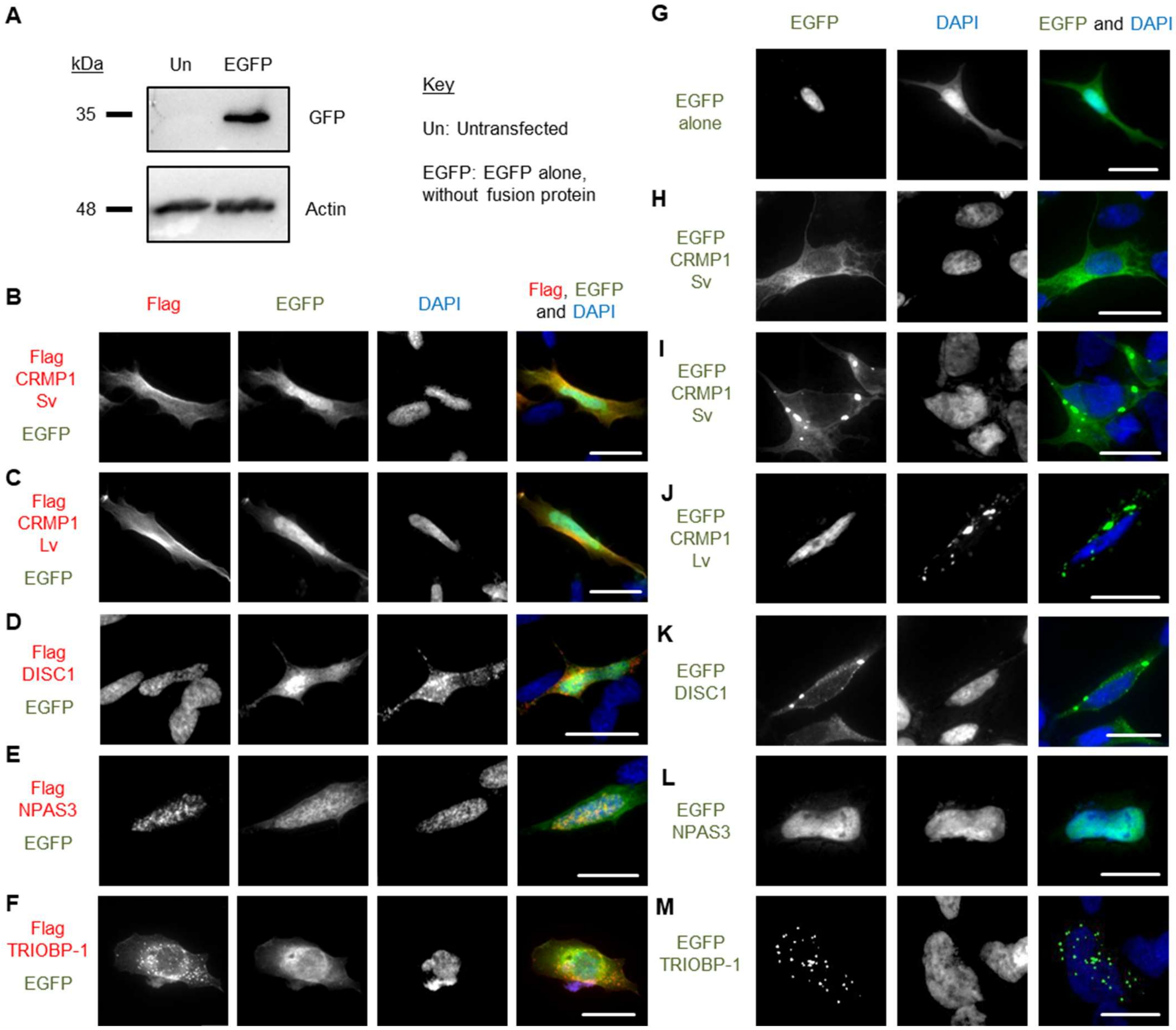

Figure S4. Expression of plasmids, in SH-SY5Y cells unless otherwise stated. **(A)** Western blot, showing expression of a construct encoding EGFP alone, with no fusion protein, in HEK293 cells. **(B-F)** Expression of Flag-tagged proteins (the same vectors as in figure 4C-G of the main text) together with EGFP alone: CRMP1 Sv (B), CRMP1 Lv (C), DISC1 (D), NPAS3 (E) and TRIOBP-1 (F). There are no obvious effects on expression patterns of the Flag tagged proteins. **(G)** Expression of EGFP alone. **(H-M)** Expression of EGFP-fused proteins, CRMP1 Sv with no aggregation (H, as seen in most cells), CRMP1 Sv with aggregation (I, minority of cells), CRMP1 Lv (J), DISC1 (K), NPAS3 (L) and TRIOBP-1 (M). Except for some EGFP-CRMP1 Sv & Lv cells showing aggregation (I, J), all constructs showed similar expression patterns to their Flag-tagged counterparts. All images are typical of three independent experiments. Scale bars represent 10 µm.

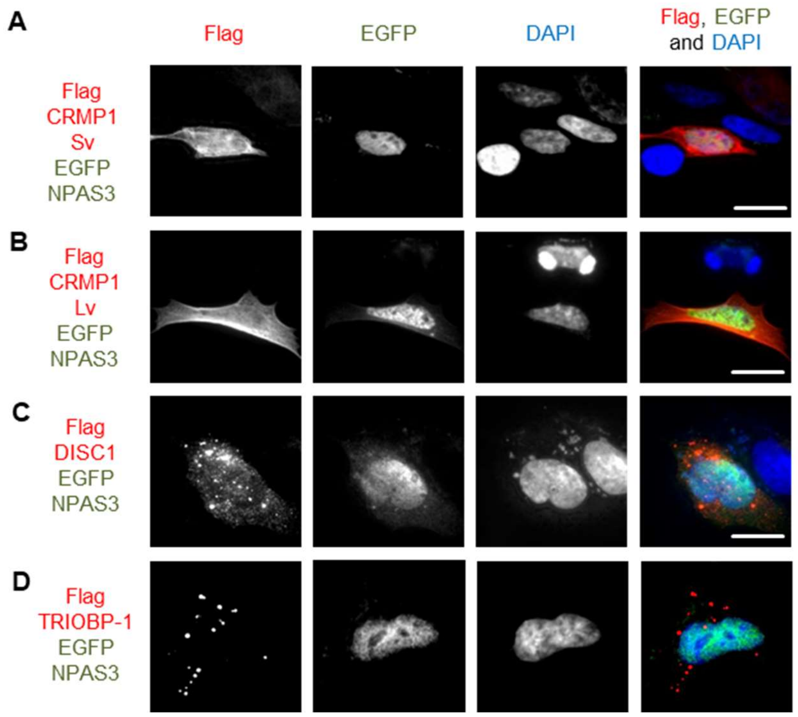

Figure S5. Expression of plasmids in SH-SY5Y cells, in experiments using reciprocal plasmid vectors from those in the main text. **(A)** Flag-tagged CRMP1 Sv and EGFP-fused NPAS3, neither aggregating, reciprocal experiment to that in figure 4H. **(B)** Flag-tagged CRMP1 Lv and EGFP-fused NPAS3, neither aggregating, reciprocal experiment to that in figure 4I. **(C)** Flag-tagged DISC1 and EGFP-fused NPAS3, only DISC1 is aggregating, reciprocal experiment to that in figure 4J. **(D)** Flag-tagged TRIOBP-1 and EGFP-fused NPAS3, only TRIOBP-1 is aggregating, reciprocal experiment to that in figure 4K. All images are typical of three independent experiments. Scale bars represent 10 µm.

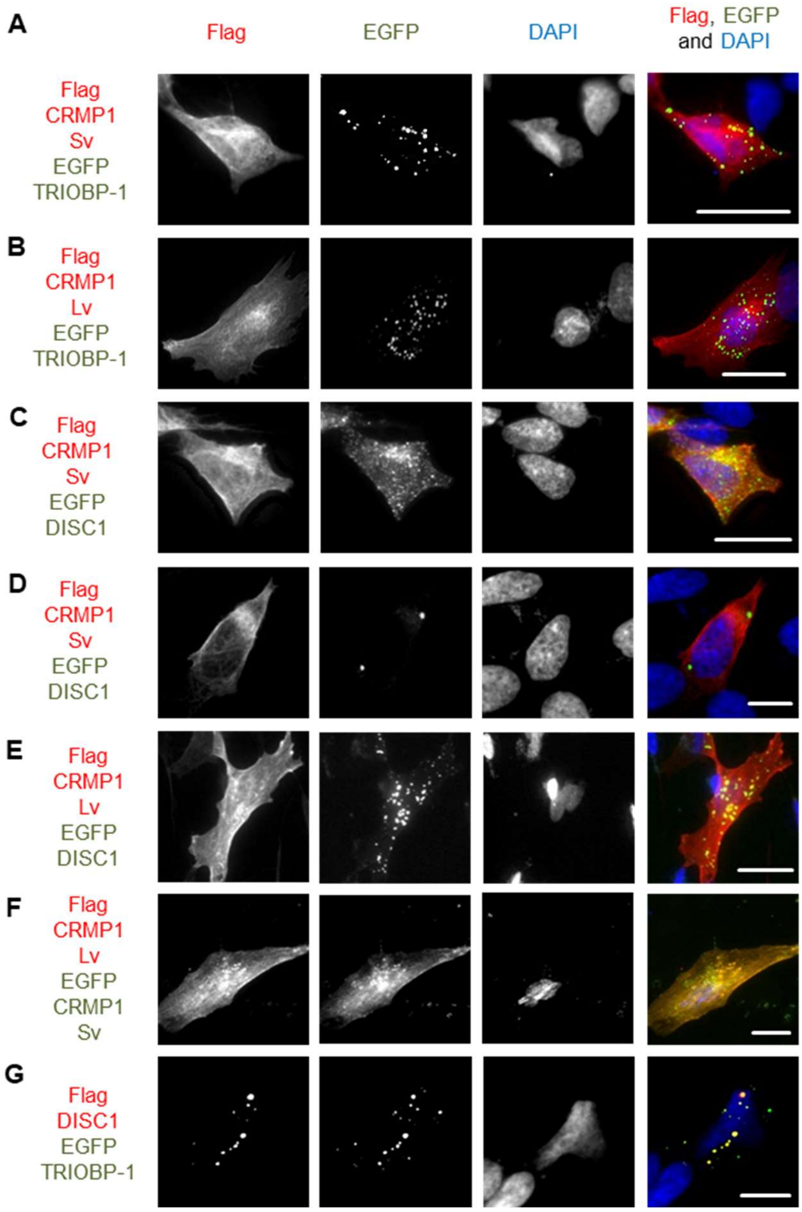

Figure S6. Expression of plasmids in SH-SY5Y cells, in experiments using reciprocal plasmid vectors from those in the main text. **(A)** Flag-tagged CRMP-1 Sv and EGFP-fused TRIOBP-1, only TRIOBP-1 aggregates, reciprocal experiment to that in figure 5A. **(B)** Flag-tagged CRMP-1 Lv and EGFP-fused TRIOBP-1, only TRIOBP-1 aggregates, reciprocal experiment to that in figure 5B. **(C-D)** Flag-tagged CRMP-1 Sv and EGFP-fused DISC1, example of cell with co-aggregation (C) and DISC1 aggregation only (D), reciprocal experiment to that in figure 5C. **(E)** Flag-tagged CRMP-1 Lv and EGFP-fused DISC1 with some co-aggregation, reciprocal experiment to that in figure 5D. **(F)** Flag-tagged CRMP1 Lv and EGFP-fused CRMP1 Sv, with co-aggregation seen, reciprocal experiment to that in figure 5E. **(G)** Flag-tagged DISC1 and EGFP-fused TRIOBP-1, with extensive co-aggregation seen, reciprocal experiment to that in figure 5F. All images are typical of three independent experiments. Scale bars represent 10 µm.

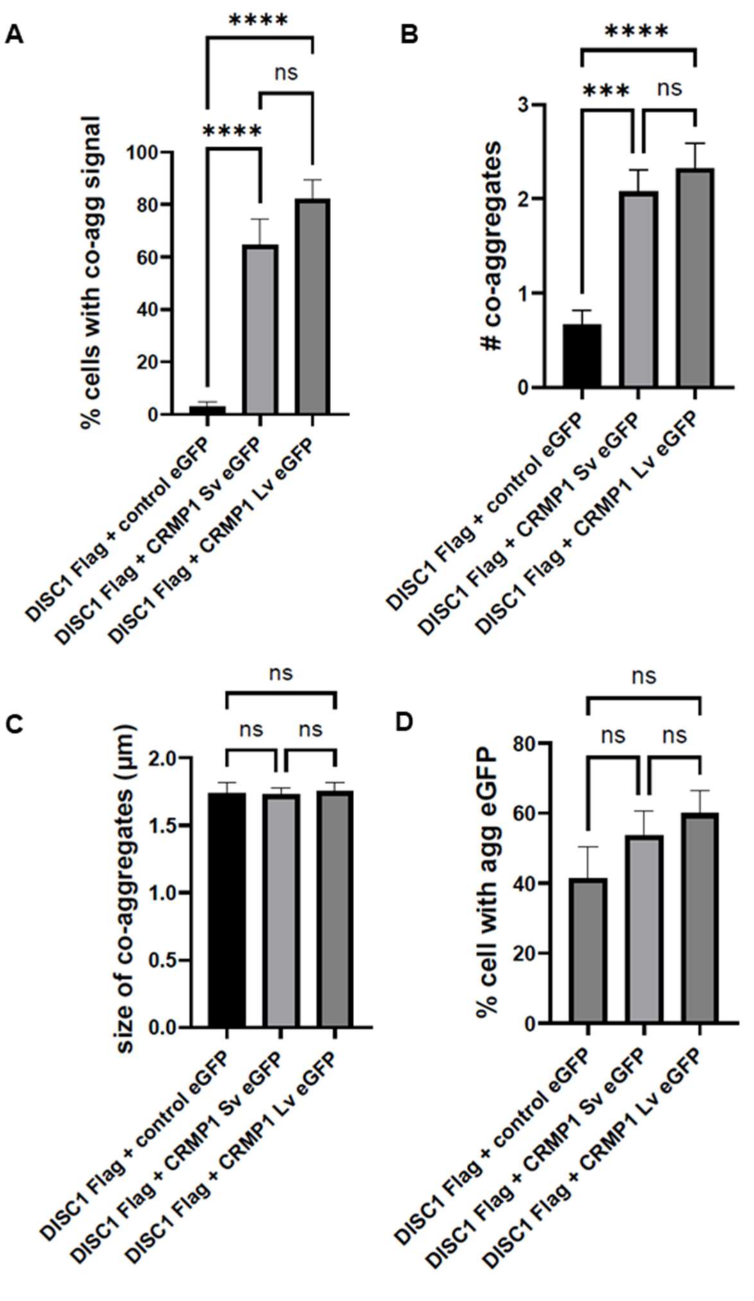

Figure S7. Additional quantification of DISC1 and CRMP1 aggregation in a co-aggregation blinded immunofluorescent microscopy assay (see figure 6). SH-SY5Y cells were transfected with Flag-DISC1 and one of EGFP, EGFP-CRMP1 Sv or EGFP-CRMP1 Lv. Results are an average of 10 coverslips per plasmid combination (9 in the case of DISC1 + EGFP), with 10 transfected cells examined per coverslip (or as many transfected cells as could be found). **(A)** Mean percentage of successfully transfected cells in which co-aggregation of Flag and EGFP seem to occur, defined as co-localisation at a punctate structure at least 1 µm in diameter. **(B)** Number of distinct co-aggregates in such cells. **(C)** Mean size of co-aggregates in such cells. **(D)** Percentage of successfully transfected cells in which aggregates of EGFP were seen (regardless of DISC1 aggregation status). All statistics are one-way ANOVA: ****: $p < 0.0001$, ***: $p < 0.001$, ns: not significant.

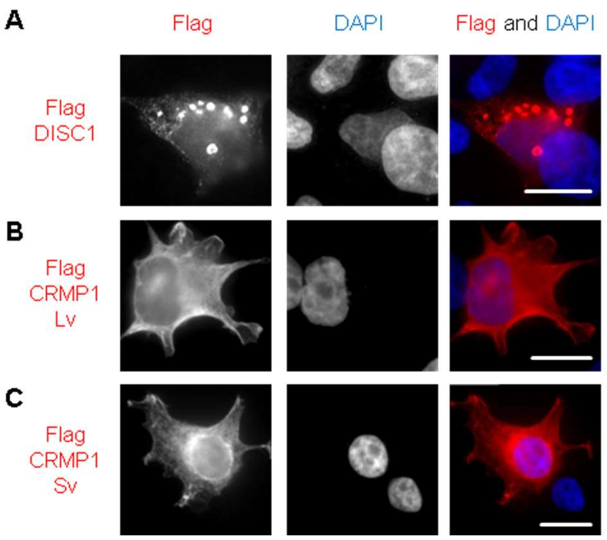

Figure S8. Expression patterns of Flag-tagged proteins expressed in HEK293 closely matches the patterns seen when the same proteins are expressed in SH-SY5Y (as shown in figure 4). **(A)** DISC1. **(B)** CRMP-1 Lv. **(C)** CRMP-1 Sv. Scale bars represent 10 µm.

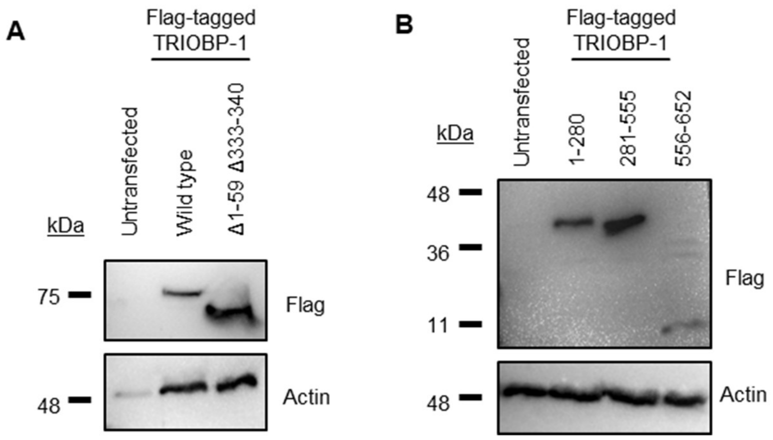

Figure S9. Western blots of HEK293 cells expressing the TRIOBP-1 constructs. **(A)** Expression of wild type (full length) TRIOBP-1 compared to a mutant version with two deletions, as used in figures 6B-C. **(B)** Expression of individual domains of TRIOBP-1, as used in figures 6D-I.

Appendix S1 - Amino acid of proteins expressed using plasmid vectors in this study

Amino acid sequences of proteins expressed from plasmid vectors in this study, as confirmed by sequencing. Numbers in brackets refer to table S1.

In all instances, amino acids listed in blue indicate the endogenous protein sequence, amino acids in red indicate Flag or c-Myc tags, amino acids in green indicate an EGFP fusion protein, and amino acids in black indicate other non-endogenous residues added in the cloning process.

(11) pdcDNA-Flag-CRMP1 Sv

MDYKDDDDKAAITSLYKKAGFKGTNSVDMSYQGKKSIPHITSDRLLIKGGRIINDDQSLYADVYLEDGLIKQIGE
NLIVPGGVKTIEANGRMVIPGGIDVNTYLQKPSQGMTAADDFFQGTRAALVGGTTMIIDHVVPEPGSSLLTSFEK
WHEAADTKSCCDYSLHVDITSWYDGVREELEVLVQDKGVNSFQVYMAYKDVYQMSDSQLYEAFTFLKGLGAVILV
HAENGDLIAQEQKRILEMGITGPEGHALSRPEELEAEAVFRAITIAGRINCPVYITKVMSKSAADIIALARKKGP
LVFGEPIAASLGTDGTHYWSKNWAKAAAFVTSPPLSPDPTTPDYLTSLLACGDLQVTGSGHCPYSTAQKAVGKDN
FTLIPEGVNGIEERMTVVWDKAVATGKMDENQFVAVTSTNAAKIFNLYPRKGRIAVGSDADVVIWDPDKLKTITA
KSHKSAVEYNIFEGMECHGSPLVVISQGKIVFEDGNINVNKGMGRFIPRKAFPEHLYQRVKIRNKVFGLQGVSRG
MYDGPVYEVPATPKYATPAPSAKSSPSKHQPPPIRNLHQSNFSLSGAQIDDNNPRRTGHRIVAPPGGRSNITSLG
*

(12) pdcDNA-Flag-CRMP1 Lv

MDYKDDDDKAAITSLYKKAGFKGTNSVDMADRRRAWNTEDDLPVYLARPGSAAQTPRQKYGGMFAAVEGAYENKT
IDFDAYSVGRRGSARTPRSAGRPDAVGLPGPGGSEDTASDVSEPSGSAVSSPGERDERPPTLRIRRPAPRDLPLG
RDNGQSDRLLIKGGRIINDDQSLYADVYLEDGLIKQIGENLIVPGGVKTIEANGRMVIPGGIDVNTYLQKPSQGM
TAADDFFQGTRAALVGGTTMIIDHVVPEPGSSLLTSFEKWHEAADTKSCCDYSLHVDITSWYDGVREELEVLVQD
KGVNSFQVYMAYKDVYQMSDSQLYEAFTFLKGLGAVILVHAENGDLIAQEQKRILEMGITGPEGHALSRPEELEA
EAVFRAITIAGRINCPVYITKVMSKSAADIIALARKKGPLVFGEPIAASLGTDGTHYWSKNWAKAAAFVTSPPLS
PDPTTPDYLTSLLACGDLQVTGSGHCPYSTAQKAVGKDNFTLIPEGVNGIEERMTVVWDKAVATGKMDENQFVAV
TSTNAAKIFNLYPRKGRIAVGSDADVVIWDPDKLKTITAKSHKSAVEYNIFEGMECHGSPLVVISQGKIVFEDGN
INVNKGMGRFIPRKAFPEHLYQRVKIRNKVFGLQGVSRGMYDGPVYEVPATPKYATPAPSAKSSPSKHQPPPIRN
LHQSNFSLSGAQIDDNNPRRTGHRIVAPPGGRSNITSLG*

(13) pdcDNA-Flag-DISC1

MDYKDDDDKAAITSLYKKAGFKGTNSVDMPGGGPQGAPAAAGGGGVSHRAGSRDCLPPAACFRRRRLARRPGYMR
SSTGPGIGFLSPAVGTLFRFPGGVSGEESHHSESRARQCGLDSRGLLVRSPVSKSAAAPTVTSVRGTSAHFGIQL
RGGTRLPDRLSWPCGPGSAGWQQEFAAMDSSETLDASWEAACSDGARRVRAAGSLPSAELSSNSCSPGCGPEVPP
TPPGSHSAFTSSFSFIRLSLGSAGERGEAEGCPPSREAESHCQSPQEMGAKAASLDGPHEDPRCLSQPFSLLATR
VSADLAQAARNSSRPERDMHSLPDMDPGSSSSLDPSLAGCGGDGSSGSGDAHSWDTLLRKWEPVLRDCLLRNRRQ
MEVISLRLKLQKLQEDAVENDDYDKAETLQQRLEDLEQEKISLHFQLPSRQPALSSFLGHLAAQVQAALRRGATQ
QASGDDTHTPLRMEPRLLEPTAQDSLHVSITRRDWLLQEKQQLQKEIEALQARMFVLEAKDQQLRREIEEQEQQL
QWQGCDLTPLVGQLSLGQLQEVSKALQDTLASAGQIPFHAEPPETIRSLQERIKSLNLSLKEITTKVCMSEKFCS
TLRKKVNDIETQLPALLEAKMHAISGNHFWTAKDLTEEIRSLTSEREGLEGLLSKLLVLSSRNVKKLGSVKEDYN
RLRREVEHQETAYETSVKENTMKYMETLKNKLCSCKCPLLGKVWEADLEACRLLIQSLQLQEARGSLSVEDERQM
DDLEGAAPPIPPRLHSEDKRKTPLKESYILSAELGEKCEDIGKKLLYLEDQLHTAIHSHDEDLIQSLRRELQMVK
ETLQAMILQLQPAKEAGEREAAASCMTAGVHEAQAYPTFLYKVVIEQKLISEEDLN*

(14) pdcDNA-Flag-NPAS3

MDYKDDDDKAAITSLYKKVGMAPTKPSFQQDPSRRERLQALRKEKSRDAARSRRGKENFEFYELAKLLPLPAAIT
SQLDKASIIRLTISYLKMRDFANQGDPPWNLRMEGPPPNTSVKGIQMWKSELCMRKTPCEVIGAQRRRSPSALAI
EVFEAHLGSHILQSLDGFVFALNQEGKFLYISETVSIYLGLSQVELTGSSVFDYVHPGDHVEMAEQLGMKLPPGR
GLLSQGTAEDGASSASSSSQSETPEPVESTSPSLLTTDNTLERSFFIRMKSTLTKRGVHIKSSGYKVIHITGRLR
LRVSLSHGRTVPSQIMGLVVVAHALPPPTINEVRIDCHMFVTRVNMDLNIIYCENRISDYMDLTPVDIVGKRCYH
FIHAEDVEGIRHSHLDLLNKGQCVTKYYRWMQKNGGYIWIQSSATIAINAKNANEKNIIWVNYLLSNPEYKDTPM
DIAQLPHLPEKTSESSETSDSESDSKDTSGITEDNENSKSDEKGNQSENSEDPEPDRKKSGNACDNDMNCNDDGH
SSSNPDSRDSDDSFEHSDFENPKAGEDGFGALGAMQIKVERYVESESDLRLQNCESLTSDSAKDSDSAGEAGAQA
SSKHQKRKKRRKRQKGGSASRRRLSSASSPGGLDAGLVEPPRLLSSPNSASVLKIKTEISEPINFDNDSSIWNYP
PNREISRNESPYSMTKPPSSEHFPSPQGGGGGGGGGGGLHVAIPDSVLTPPGADGAAARKTQFGASATAALAPVA
SDPLSPPLSASPRDKHPGNGGGGGGGGGGAGGGGPSASNSLLYTGDLEALQRLQAGNVVLPLVHRVTGTLAATST
AAQRVYTTGTIRYAPAEVTLAMQSNLLPNAHAVNFVDVNSPGFGLDPKTPMEMLYHHVHRLNMSGPFGGAVSAAS
LTQMPAGNVFTTAEGLFSTLPFPVYSNGIHAAQTLERKEDYPTFLYKVVIEQKLISEEDLN*

(15) pdcDNA-Flag-TRIOBP-1 (wild type)

MDYKDDDDKAAITSLYKKAGFKGTNSVDMGGWKGPGQRRGKEGPEARRRAAERGGGGGGGGVPAPRSPAREPRPR
SCLLLPPPWGAAMTPDLLNFKKGWMSILDEPGEPPSPSLTTTSTSQWKKHWFVLTDSSLKYYRDSTAEEADELDG
EIDLRSCTDVTEYAVQRNYGFQIHTKDAVYTLSAMTSGIRRNWIEALRKTVRPTSAPDVTKLSDSNKENALHSYS
TQKGPLKAGEQRAGSEVISRGGPRKADGQRQALDYVELSPLTQASPQRARTPARTPDRLAKQEELERDLAQRSEE
RRKWFEATDSRTPEVPAGEGPRRGLGAPLTEDQQNRLSEEIEKKWQELEKLPLRENKRVPLTALLNQSRGERRGP
PSDGHEALEKEVQALRAQLEAWRLQGEAPQSALRSQEDGHIPPGYISQEACERSLAEMESSHQQVMEELQRHHER
ELQRLQQEKEWLLAEETAATASAIEAMKKAYQEELSRELSKTRSLQQGPDGLRKQHQSDVEALKRELQVLSEQYS
QKCLEIGALMRQAEEREHTLRRCQQEGQELLRHNQELHGRLSEEIDQLRGFIASQGMGNGCGRSNERSSCELEVL
LRVKENELQYLKKEVQCLRDELQMMQKDKRFTSGKYQDVYVELSHIKTRSEREIEQLKEHLRLAMAALQEKESMR
NSLAE*

(16) pdcDNA-Flag-TRIOBP-1 Δ1-59Δ333-340

MDYKDDDDKAAITSLYKKAGFKGTNSVDMTPDLLNFKKGWMSILDEPGEPPSPSLTTTSTSQWKKHWFVLTDSSL
KYYRDSTAEEADELDGEIDLRSCTDVTEYAVQRNYGFQIHTKDAVYTLSAMTSGIRRNWIEALRKTVRPTSAPDV
TKLSDSNKENALHSYSTQKGPLKAGEQRAGSEVISRGGPRKADGQRQALDYVELSPLTQASPQRARTPARTPDRL
AKQEELERDLAQRSEERRKWFEATDSRTPEVPAGEGPRRGLGAPLTEDQQNRLSEEIEKKWQELEKLPLRENKRV
PGTRGERRGPPSDGHEALEKEVQALRAQLEAWRLQGEAPQSALRSQEDGHIPPGYISQEACERSLAEMESSHQQV
MEELQRHHERELQRLQQEKEWLLAEETAATASAIEAMKKAYQEELSRELSKTRSLQQGPDGLRKQHQSDVEALKR
ELQVLSEQYSQKCLEIGALMRQAEEREHTLRRCQQEGQELLRHNQELHGRLSEEIDQLRGFIASQGMGNGCGRSN
ERSSCELEVLLRVKENELQYLKKEVQCLRDELQMMQKDKRFTSGKYQDVYVELSHIKTRSEREIEQLKEHLRLAM
AALQEKESMRNSLAE*

(17) pdcDNA-Flag-TRIOBP-1 1-280

MDYKDDDDKAAITSLYKKAGFAATMGGWKGPGQRRGKEGPEARRRAAERGGGGGGGGVPAPRSPAREPRPR
SCLLLPPPWGAAMTPDLLNFKKGWMSILDEPGEPPSPSLTTTSTSQWKKHWFVLTDSSLKYYRDSTAEEAD
ELDGEIDLRSCTDVTEYAVQRNYGFQIHTKDAVYTLSAMTSGIRRNWIEALRKTVRPTSAPDVTKLSDSNK
ENALHSYSTQKGPLKAGEQRAGSEVISRGGPRKADGQRQALDYVELSPLTQASPQRARTPARTPDRLAKQE
ELERDLAQRSEERRKWFEAT*

(18) pdcDNA-Flag-TRIOBP-1 281-555

MDYKDDDDKAAITSLYKKAGFAATMDSRTPEVPAGEGPRRGLGAPLTEDQQNRLSEEIEKKWQELEKLPLRENKR
VPLTALLNQSRGERRGPPSDGHEALEKEVQALRAQLEAWRLQGEAPQSALRSQEDGHIPPGYISQEACERSLAEM
ESSHQQVMEELQRHHERELQRLQQEKEWLLAEETAATASAIEAMKKAYQEELSRELSKTRSLQQGPDGLRKQHQS
DVEALKRELQVLSEQYSQKCLEIGALMRQAEEREHTLRRCQQEGQELLRHNQELHGRLSEEIDQLRGFIASQGMG
*

(19) pdcDNA-Flag-TRIOBP-1 556-652

MDYKDDDDKAAITSLYKKAGFAATMNGCGRSNERSSCELEVLLRVKENELQYLKKEVQCLRDELQMMQKD
KRFTSGKYQDVYVELSHIKTRSEREIEQLKEHLRLAMAALQEKESMRNSLAE*

(20) pDEST-CMV-N-EGFP (empty control)

MVSKGEELFTGVVPILVELDGDVNGHKFSVSGEGEGDATYGKLTLKFICTTGKLPVPWPTLVTTLTYGVQCFSRY
PDHMKQHDFFKSAMPEGYVQERTIFFKDDGNYKTRAEVKFEGDTLVNRIELKGIDFKEDGNILGHKLEYNYNSHN
VYIMADKQKNGIKVNFKIRHNIEDGSVQLADHYQQNTPIGDGPVLLPDNHYLSTQSALSKDPNEKRDHMVLLEFV
TAAGITHGMDELYKAVPDRIKQVCTKKQALKEPIQSTGSGTEFAAALEISRPSFLVQSGDN*

(21) pDEST-CMV-N-EGFP-CRMP1 Sv

MVSKGEELFTGVVPILVELDGDVNGHKFSVSGEGEGDATYGKLTLKFICTTGKLPVPWPTLVTTLTYGVQCFSRY
PDHMKQHDFFKSAMPEGYVQERTIFFKDDGNYKTRAEVKFEGDTLVNRIELKGIDFKEDGNILGHKLEYNYNSHN
VYIMADKQKNGIKVNFKIRHNIEDGSVQLADHYQQNTPIGDGPVLLPDNHYLSTQSALSKDPNEKRDHMVLLEFV
TAAGITHGMDELYKAVPDRDQTSLYKKAGFKGTNSVDGMSYQGKKSIPHITSDRLLIKGGRIINDDQSLYADVYL
EDGLIKQIGENLIVPGGVKTIEANGRMVIPGGIDVNTYLQKPSQGMTAADDFFQGTRAALVGGTTMIIDHVVPEP
GSSLLTSFEKWHEAADTKSCCDYSLHVDITSWYDGVREELEVLVQDKGVNSFQVYMAYKDVYQMSDSQLYEAFTF
LKGLGAVILVHAENGDLIAQEQKRILEMGITGPEGHALSRPEELEAEAVFRAITIAGRINCPVYITKVMSKSAAD
IIALARKKGPLVFGEPIAASLGTDGTHYWSKNWAKAAAFVTSPPLSPDPTTPDYLTSLLACGDLQVTGSGHCPYS
TAQKAVGKDNFTLIPEGVNGIEERMTVVWDKAVATGKMDENQFVAVTSTNAAKIFNLYPRKGRIAVGSDADVVIW
DPDKLKTITAKSHKSAVEYNIFEGMECHGSPLVVISQGKIVFEDGNINVNKGMGRFIPRKAFPEHLYQRVKIRNK
VFGLQGVSRGMYDGPVYEVPATPKYATPAPSAKSSPSKHQPPPIRNLHQSNFSLSGAQIDDNNPRRTGHRIVAPP
GGRSNITSLG*

(22) pDEST-CMV-N-EGFP-CRMP1 Lv

MVSKGEELFTGVVPILVELDGDVNGHKFSVSGEGEGDATYGKLTLKFICTTGKLPVPWPTLVTTLTYGVQCFSRY
PDHMKQHDFFKSAMPEGYVQERTIFFKDDGNYKTRAEVKFEGDTLVNRIELKGIDFKEDGNILGHKLEYNYNSHN
VYIMADKQKNGIKVNFKIRHNIEDGSVQLADHYQQNTPIGDGPVLLPDNHYLSTQSALSKDPNEKRDHMVLLEFV
TAAGITHGMDELYKAVPDRDQTSLYKKAGFKGTNSVDGMADRRRAWNTEDDLPVYLARPGSAAQTPRQKYGGMFA
AVEGAYENKTIDFDAYSVGRRGSARTPRSAGRPDAVGLPGPGGSEDTASDVSEPSGSAVSSPGERDERPPTLRIR
RPAPRDLPLGRDNGQSDRLLIKGGRIINDDQSLYADVYLEDGLIKQIGENLIVPGGVKTIEANGRMVIPGGIDVN
TYLQKPSQGMTAADDFFQGTRAALVGGTTMIIDHVVPEPGSSLLTSFEKWHEAADTKSCCDYSLHVDITSWYDGV
REELEVLVQDKGVNSFQVYMAYKDVYQMSDSQLYEAFTFLKGLGAVILVHAENGDLIAQEQKRILEMGITGPEGH
ALSRPEELEAEAVFRAITIAGRINCPVYITKVMSKSAADIIALARKKGPLVFGEPIAASLGTDGTHYWSKNWAKA
AAFVTSPPLSPDPTTPDYLTSLLACGDLQVTGSGHCPYSTAQKAVGKDNFTLIPEGVNGIEERMTVVWDKAVATG
KMDENQFVAVTSTNAAKIFNLYPRKGRIAVGSDADVVIWDPDKLKTITAKSHKSAVEYNIFEGMECHGSPLVVIS
QGKIVFEDGNINVNKGMGRFIPRKAFPEHLYQRVKIRNKVFGLQGVSRGMYDGPVYEVPATPKYATPAPSAKSSP
SKHQPPPIRNLHQSNFSLSGAQIDDNNPRRTGHRIVAPPGGRSNITSLG*

(23) pDEST-CMV-N-EGFP-DISC1

MVSKGEELFTGVVPILVELDGDVNGHKFSVSGEGEGDATYGKLTLKFICTTGKLPVPWPTLVTTLTYGVQCFSRY
PDHMKQHDFFKSAMPEGYVQERTIFFKDDGNYKTRAEVKFEGDTLVNRIELKGIDFKEDGNILGHKLEYNYNSHN
VYIMADKQKNGIKVNFKIRHNIEDGSVQLADHYQQNTPIGDGPVLLPDNHYLSTQSALSKDPNEKRDHMVLLEFV
TAAGITHGMDELYKAVPDRDQTSLYKKVGTMPGGGPQGAPAAAGGGGVSHRAGSRDCLPPAACFRRRRLARRPGY
MRSSTGPGIGFLSPAVGTLFRFPGGVSGEESHHSESRARQCGLDSRGLLVRSPVSKSAAAPTVTSVRGTSAHFGI
QLRGGTRLPDRLSWPCGPGSAGWQQEFAAMDSSETLDASWEAACSDGARRVRAAGSLPSAELSSNSCSPGCGPEV
PPTPPGSHSAFTSSFSFIRLSLGSAGERGEAEGCPPSREAESHCQSPQEMGAKAASLDGPHEDPRCLSQPFSLLA
TRVSADLAQAARNSSRPERDMHSLPDMDPGSSSSLDPSLAGCGGDGSSGSGDAHSWDTLLRKWEPVLRDCLLRNR
RQMEVISLRLKLQKLQEDAVENDDYDKAETLQQRLEDLEQEKISLHFQLPSRQPALSSFLGHLAAQVQAALRRGA
TQQASGDDTHTPLRMEPRLLEPTAQDSLHVSITRRDWLLQEKQQLQKEIEALQARMFVLEAKDQQLRREIEEQEQ
QLQWQGCDLTPLVGQLSLGQLQEVSKALQDTLASAGQIPFHAEPPETIRSLQERIKSLNLSLKEITTKVCMSEKF
CSTLRKKVNDIETQLPALLEAKMHAISGNHFWTAKDLTEEIRSLTSEREGLEGLLSKLLVLSSRNVKKLGSVKED
YNRLRREVEHQETAYETSVKENTMKYMETLKNKLCSCKCPLLGKVWEADLEACRLLIQSLQLQEARGSLSVEDER
QMDDLEGAAPPIPPRLHSEDKRKTPLKESYILSAELGEKCEDIGKKLLYLEDQLHTAIHSHDEDLIQSLRRELQM
VKETLQAMILQLQPAKEAGEREAAASCMTAGVHEAQADPAFLYKVVIIN*

(24) pDEST-CMV-N-EGFP-NPAS3

MVSKGEELFTGVVPILVELDGDVNGHKFSVSGEGEGDATYGKLTLKFICTTGKLPVPWPTLVTTLTYGVQCFSRY
PDHMKQHDFFKSAMPEGYVQERTIFFKDDGNYKTRAEVKFEGDTLVNRIELKGIDFKEDGNILGHKLEYNYNSHN
VYIMADKQKNGIKVNFKIRHNIEDGSVQLADHYQQNTPIGDGPVLLPDNHYLSTQSALSKDPNEKRDHMVLLEFV
TAAGITHGMDELYKAVPDRDQTSLYKKVGTAVPDRIKQVCTKKQKGRQGPPMAPTKPSFQQDPSRRERLQALRKE
KSRDAARSRRGKENFEFYELAKLLPLPAAITSQLDKASIIRLTISYLKMRDFANQGDPPWNLRMEGPPPNTSVKG
IQMWKSELCMRKTPCEVIGAQRRRSPSALAIEVFEAHLGSHILQSLDGFVFALNQEGKFLYISETVSIYLGLSQV
ELTGSSVFDYVHPGDHVEMAEQLGMKLPPGRGLLSQGTAEDGASSASSSSQSETPEPVESTSPSLLTTDNTLERS
FFIRMKSTLTKRGVHIKSSGYKVIHITGRLRLRVSLSHGRTVPSQIMGLVVVAHALPPPTINEVRIDCHMFVTRV
NMDLNIIYCENRISDYMDLTPVDIVGKRCYHFIHAEDVEGIRHSHLDLLNKGQCVTKYYRWMQKNGGYIWIQSSA
TIAINAKNANEKNIIWVNYLLSNPEYKDTPMDIAQLPHLPEKTSESSETSDSESDSKDTSGITEDNENSKSDEKG
NQSENSEDPEPDRKKSGNACDNDMNCNDDGHSSSNPDSRDSDDSFEHSDFENPKAGEDGFGALGAMQIKVERYVE
SESDLRLQNCESLTSDSAKDSDSAGEAGAQASSKHQKRKKRRKRQKGGSASRRRLSSASSPGGLDAGLVEPPRLL
SSPNSASVLKIKTEISEPINFDNDSSIWNYPPNREISRNESPYSMTKPPSSEHFPSPQGGGGGGGGGGGLHVAIP
DSVLTPPGADGAAARKTQFGASATAALAPVASDPLSPPLSASPRDKHPGNGGGGGGGGGGAGGGGPSASNSLLYT
GDLEALQRLQAGNVVLPLVHRVTGTLAATSTAAQRVYTTGTIRYAPAEVTLAMQSNLLPNAHAVNFVDVNSPGFG
LDPKTPMEMLYHHVHRLNMSGPFGGAVSAASLTQMPAGNVFTTAEGLFSTLPFPVYSNGIHAAQTLERKEDDPAF
LYKVVIIN*

(25) pDEST-CMV-N-EGFP-TRIOBP-1

MVSKGEELFTGVVPILVELDGDVNGHKFSVSGEGEGDATYGKLTLKFICTTGKLPVPWPTLVTTLTYGVQCFSRY
PDHMKQHDFFKSAMPEGYVQERTIFFKDDGNYKTRAEVKFEGDTLVNRIELKGIDFKEDGNILGHKLEYNYNSHN
VYIMADKQKNGIKVNFKIRHNIEDGSVQLADHYQQNTPIGDGPVLLPDNHYLSTQSALSKDPNEKRDHMVLLEFV
TAAGITHGMDELYKAVPDRDQTSLYKKAGFKGTNSVDMGGWKGPGQRRGKEGPEARRRAAERGGGGGGGGVPAPR
SPAREPRPRSCLLLPPPWGAAMTPDLLNFKKGWMSILDEPGEPPSPSLTTTSTSQWKKHWFVLTDSSLKYYRDST
AEEADELDGEIDLRSCTDVTEYAVQRNYGFQIHTKDAVYTLSAMTSGIRRNWIEALRKTVRPTSAPDVTKLSDSN
KENALHSYSTQKGPLKAGEQRAGSEVISRGGPRKADGQRQALDYVELSPLTQASPQRARTPARTPDRLAKQEELE
RDLAQRSEERRKWFEATDSRTPEVPAGEGPRRGLGAPLTEDQQNRLSEEIEKKWQELEKLPLRENKRVPLTALLN
QSRGERRGPPSDGHEALEKEVQALRAQLEAWRLQGEAPQSALRSQEDGHIPPGYISQEACERSLAEMESSHQQVM
EELQRHHERELQRLQQEKEWLLAEETAATASAIEAMKKAYQEELSRELSKTRSLQQGPDGLRKQHQSDVEALKRE
LQVLSEQYSQKCLEIGALMRQAEEREHTLRRCQQEGQELLRHNQELHGRLSEEIDQLRGFIASQGMGNGCGRSNE
RSSCELEVLLRVKENELQYLKKEVQCLRDELQMMQKDKRFTSGKYQDVYVELSHIKTRSEREIEQLKEHLRLAMA
ALQEKESMRNSLAE*
